# Epithelial interferon-stimulated gene programs modulate chemotherapy sensitivity in colorectal cancer via *EPSTI1*

**DOI:** 10.1038/s41388-026-03957-9

**Published:** 2026-08-21

**Authors:** Erdogan Pekcan Erkan, Emmi Hämäläinen, Julia Kolikova, Tiia Nikupaavola, Karishma Sajnani, Kornelia Kuc, Kalle Ojala, Meiju Kukkonen, Ismaïl Hermelo, Antti Kurttila, Selja Koskensalo, Timo Tarvainen, Alli Leppä, Ilona Keränen, Carola Haapamäki, Essi Karjalainen, Monika Carpelan-Holmström, Laura Renkonen-Sinisalo, Laura Koskenvuo, Pauli Puolakkainen, Jukka-Pekka Mecklin, Juha P. Väyrynen, Elina Virolainen, iCAN, Erdogan Pekcan Erkan, Emmi Hämäläinen, Julia Kolikova, Tiia Nikupaavola, Kalle Ojala, Selja Koskensalo, Timo Tarvainen, Alli Leppä, Ilona Keränen, Carola Haapamäki, Essi Karjalainen, Monika Carpelan-Holmström, Laura Renkonen-Sinisalo, Laura Koskenvuo, Pauli Puolakkainen, Elina Virolainen, Lauri A. Aaltonen, Anna Lepistö, Ari Ristimäki, Toni T. Seppälä, Matti Nykter, Lauri A. Aaltonen, Alfonso Urbanucci, Anna Lepistö, Ari Ristimäki, Toni T. Seppälä

**Affiliations:** 1 Center for Cancer Eradication Research, Faculty of Medicine and Health Technology, Tampere University and TAYS Cancer Centre, Tampere University Hospital, Tampere, Finland; 2 Department NEON-S, Faculty of Medicine and Health Technology, Tampere University, Tampere, Finland; 3 Applied Tumor Genomics Research Program, Research Programs Unit, University of Helsinki, Helsinki, Finland; 4 Department MEDI, Faculty of Medicine and Health Technology, Tampere University, Tampere, Finland; 5 Department of Abdominal Surgery, Helsinki University Hospital and University of Helsinki, Helsinki, Finland; 6 Faculty of Medicine and Health Technology, Tampere University, Tampere, Finland; 7 Sport and Health Sciences, Jyväskylä University, Jyväskylä, Finland; 8 Department of Education and Research, Wellbeing Services County of Central Finland, Jyväskylä, Finland; 9 Translational Medicine Research Unit, Medical Research Center Oulu, Oulu University Hospital and University of Oulu, Oulu, Finland; 10 Department of Pathology, Helsinki University Hospital, Helsinki, Finland; 11 Department of Tumor Biology, Institute for Cancer Research, Oslo University Hospital, Oslo, Norway; 12 Finnish Cancer Institute, Helsinki, Finland; 13 Department of Gastroenterology and Alimentary Tract Surgery, Tampere University Hospital, Tampere, Finland; 14 Present Address: Department of Neurosurgery, Tampere University Hospital and Tampere University, Tampere, Finland

**Keywords:** Colorectal cancer, Transcriptomics, Sequencing, Cancer models

## Abstract

Although chemotherapy in colorectal cancer (CRC) primarily targets epithelial tumor cells, the epithelial cell-intrinsic mechanisms underlying heterogeneous treatment responses remain poorly understood. We integrated the genomic, transcriptomic, and in vitro drug response profiles of CRC patient-derived tumor organoids (PDTOs) with functional assays to identify epithelial cell-intrinsic determinants of chemotherapy response. An epithelial interferon-stimulated gene (ISG) program, reflecting JAK-STAT pathway activity, was associated with reduced chemosensitivity in PDTOs. Single-cell RNA sequencing data of CRC tumors before and after neoadjuvant chemotherapy showed persistent epithelial ISG expression, with *EPSTI1* notably enriched in tumors with incomplete pathological responses. Functional studies demonstrated that *EPSTI1* knockdown reduced CRC cell viability and enhanced chemosensitivity. Pharmacologic JAK-STAT inhibition with ruxolitinib suppressed ISG expression and attenuated chemotherapy-induced ISG upregulation. These findings highlight the utility of PDTOs for studying tumor-intrinsic mechanisms of treatment response and identify *EPSTI1* as an epithelial modulator of chemosensitivity.

## Introduction

Colorectal cancer (CRC) treatment guidelines offer limited stratification based on tumor molecular features, even though patients with CRC can exhibit heterogeneous clinical responses to standard-of-care chemotherapy. Patient-derived tumor organoids (PDTOs), primarily composed of epithelial cells [1], have emerged as preclinical models that are particularly suitable for studying epithelial cell-specific features because they accurately recapitulate the histopathological and genomic characteristics of tumor tissues they are derived from [2–4]. In functional precision oncology, PDTOs have been used to study tumor progression and metastasis [5], and to evaluate and predict patient-specific responses to chemotherapy [6, 7] and immunotherapies [8]. However, these models have been underutilized to identify the molecular features of epithelial cancer cells and understand how they respond to chemotherapeutics.

Here, we integrated the genomic, transcriptomic, and drug sensitivity profiles of primary CRC tumors and matched PDTOs to investigate the epithelial cell-intrinsic mechanisms underlying heterogeneous chemotherapy responses. We identified that PDTOs with low chemosensitivity showed higher JAK-STAT pathway activity driven by epithelial-intrinsic interferon-stimulated gene (ISG) expression with both antiviral and inflammatory components. Using publicly available single cell RNA-sequencing (scRNA-seq) data of CRC tumors, we showed that ISG expression was augmented by neoadjuvant chemotherapy and was associated with incomplete pathological responses. Among these chemotherapy-modulated ISGs, we focused on epithelial stromal interaction 1 (*EPSTI1*) as a candidate effector and demonstrated its role in epithelial cell survival and chemotherapy modulation. Collectively, our results link a chemotherapy-regulated interferon (IFN) signaling program to incomplete pathological responses in CRC.

## Methods

### Patient recruitment

This study was carried out as part of the PDTO tissue acquisition protocols of the PANORG project and SYNCOPE clinical trial at the Helsinki University Hospital. Patients enrolled in the PANORG protocol also participated in the iCAN Flagship Project through the collection of biobank samples and biobank transfer of PANORG samples.

Patients were enrolled in the study protocols at their first outpatient visit to tertiary cancer care at the Meilahti Hospital (Helsinki, Finland) or Jorvi Hospital (Espoo, Finland). Both hospitals are part of the HUS Comprehensive Cancer Center. Upon written informed consent, CRC tumor tissues and germline DNA references were collected, with the primary function of molecularly characterizing primary tumors and establishing PDTO cultures.

### Tissue collection

Primary tumor tissue samples were obtained by a study surgeon either via endoscopic biopsy forceps or a conchotome before any treatment, or via biopsy from surgical specimens potentially exposed to neoadjuvant therapy. The samples were transported to the laboratory site in a transfer medium containing DMEM supplemented with 1% penicillin-streptomycin solution to establish the PDTOs. Tissue specimens were processed within 24 h of collection to minimize the risk of autolysis and tissue degradation.

Primary CRC samples for exome and transcriptome sequencing were collected to Helsinki Biobank from surgically resected specimens at the Department of Pathology. Peripheral blood mononuclear cell reference DNA samples were collected through a biobank as part of the routine laboratory workup for the participants.

### PDTO culture

Tumor tissue samples were dissociated mechanically using a P1000 pipette, followed by a 10-min enzymatic digestion with Organoid Digestion Medium. Only mechanical dissociation was used for endoscopic biopsies. Dissociated samples were centrifuged and washed three times with Organoid Wash Medium. After the final centrifugation step, the cell pellets were resuspended in Matrigel (Corning, NY, USA) and plated on a 24-well plate. The plate was incubated at 37 °C for 30 min, and 500 µL of Organoid Culture Medium supplemented with 1 µL/mL Rho-associated coiled-coil containing the protein kinase (ROCK) inhibitor Y-27632 was added. Our success rate in establishing PDTOs from surgical specimens was in agreement with earlier reports of similar sample sizes [7, 9, 10].

PDTOs were incubated at 37 °C with 21% O_2_ and 5% CO_2_ and subcultured every 7–10 days for expansion. The Wnt-activating niche factors Wnt-3a and R-spondin were not added to the Organoid Culture medium to select and support the growth of Wnt-independent tumor cells. The critical biomass for a stable organoid line for molecular and phenotypic characterization was achieved early in most cases; however, the expansion phase was continued for 4–6 passages to ensure independence from Wnt-activating supplements. In vitro drug testing was not performed before passage 5 (P5) to minimize the risk of assaying noncancerous PDTOs.

### Single-cell RNA sequencing (scRNA-seq) of PDTOs

Chromium Fixed RNA Profiling for Multiplexed Samples (10x Genomics) was used to perform single-cell RNA sequencing (scRNA-seq) on PDTOs. Briefly, PDTOs were dissociated into single cells using TrypLE Express (12605036, Gibco) and filtered through a strainer. The Chromium Next GEM Single Cell Fixed RNA Sample Preparation Kit (PN-1000414, 10x Genomics) was used to fix cells at 4 °C for 24 h. Sequencing libraries were prepared using the multiplexed workflow of the Chromium Fixed RNA Kit, Human Transcriptome (PN-1000476, 10x Genomics), and sequenced on a NovaSeq 6000 instrument (Illumina) in paired-end mode.

The cellranger multi pipeline (v.9.0.0) (10x Genomics) was used for demultiplexing, alignment, and counting. Seurat (v.5.0) R package [11] was used to create Seurat objects from the output files. In the first quality-control step, the miQC framework [12] was used to filter low-quality cells in a data-driven manner. In the second quality-control step, cells expressing fewer than 50,000 UMIs were further filtered out.

Downstream analyses were performed without integration, as the PDTO samples were processed and sequenced simultaneously. Data normalization and variance stabilization were performed with Seurat’s SCTransform function, retaining the 3000 most variable features. Dimensionality reduction was carried out by principal component analysis (PCA) using 50 components, and the first 25 principal components were used to construct a k-nearest neighbor graph. Clustering was performed using a graph-based approach across multiple resolutions (0–0.5, step size 0.1). Uniform manifold approximation and projection (UMAP) embedding was generated using the first 25 principal components with 30 neighbors and a minimum distance of 0.2.

### In vitro drug testing

5-fluorouracil (S1209, Selleckchem), oxaliplatin (S1224, Selleckchem) and irinotecan (S2217, Selleckchem) were resuspended and stored according to the manufacturer’s instructions. PDTOs were dissociated into single cells, resuspended in an Organoid Culture Medium:Matrigel (9:1) mixture, and plated in 384-well plates. Cells were allowed to recover for 48 h, and organoid formation was assessed visually. Drugs were applied using a digital liquid dispenser (Tecan D300e, Tecan, Zurich, Switzerland) across a logarithmically designed dose range spanning ten concentrations. Dimethyl sulfoxide (DMSO; max. 1%) was used as a negative control for 5-FU and irinotecan, whereas water + 0.3% Tween-20 was used as a negative control for oxaliplatin. Blasticidin (ant-bl-005, Invivogen) was used as the positive control at a final concentration of 10 µg/ml. Four technical replicates were used to correct intraplate variability. PDTOs were treated with drugs for 5 days, and the CellTiter-Glo assay (Promega, Madison, WI, US) was used to assess cell viability on day seven. The luminescence signals were measured using a multimode plate reader (FLUOstar Omega, BMG Labtech).

### Whole exome sequencing (WES)

Genomic DNA was isolated from snap-frozen PDTOs using QIAamp DNA Mini kit (#51304, QIAGEN). Library preparation was performed using Twist Library Preparation Enzymatic Fragmentation (EF) kit 2.0 with UDI indexing, utilizing 100 ng of DNA input. Target capture was performed using IDT xGEN reagents and IDT xGen™ Exome Hyb Panel v2. Libraries were sequenced on a Novaseq X instrument (25B flow cell, 2 × 150 bp). Somatic mutations were identified using a validated pipeline A minimum allele fraction of 10% was required for somatic mutation calling and variants that had a GNOMAD frequency higher than 0.5% were filtered out.

### Drug response modeling

The average luminescence signal in the blank wells was subtracted from that in each well to calculate the blank-corrected signals. Relative cell viability was calculated using the following formula:

$${Cell}\,{viability}\, \% \,=\,\frac{{Blank}\,{corrected}\,{signal}\,-\,{average}\,{positive}\,{control}\,{signal}}{{Average}\,{negative}\,{control}\,{signal}\,-\,{average}\,{positive}\,{control}\,{signal}}$$

The four-parameter logistic model is arguably the most common statistical method for identifying drug efficacy in vitro [13, 14]. It is often used regardless of the data satisfying the assumptions underlying the model, producing potentially inaccurate point estimates (IC50 or AUC) that are used for downstream analyses. Moreover, IC50 values can only be calculated if the relative cell viability is less than 50% at the highest drug concentration tested [15], a requirement that was not met by many PDTOs in our study. Therefore, we chose to use the nonparametric monotone model of the ENDS tool [13] by fitting the model to the mean responses at each dose. The AUC values were used as point estimates of in vitro drug responses.

### Quality control and drug response classification

The dose-response curves were visually evaluated. An assay was considered to have failed if the normalized cell viability was less than 50% at the lowest drug concentration or if the nonparametric model failed (e.g., a flat line was observed). Twelve PDTOs failed assays for all three drugs and were excluded from the downstream analyses. Kernel density estimation (KDE) was used on the AUC values to find two valley points to be used as thresholds for drug response classification. This approach revealed two valley points for fluorouracil, but not for irinotecan and oxaliplatin. As a secondary approach, we used the following classification for irinotecan and oxaliplatin:

**if** AUC > Q3 ~ “low sensitivity”

**elif** AUC ≤ Q1 ~ “high sensitivity”

**else**~“intermediate sensitivity”

PDTOs with missing values were classified as “Unknown”.

### Bulk RNA sequencing of PDTOs

The NucleoSpin RNA Plus (MN) kit was used to isolate total RNA from the snap-frozen cell pellets. RNA samples were eluted in nuclease-free water and stored at −80 °C. Total RNA (100 ng) was ribo-depleted using Illumina’s Stranded Total RNA Prep with Ribo-Zero Plus (20040529) and library preparation was completed using standard Illumina protocols. Indexed libraries were pooled and sequenced on an Illumina NovaSeq6000 system (Illumina, San Diego, CA, USA) with one lane of the NovaSeq S4 flow cell (2 × 150 bp).

### Preprocessing PDTO bulk RNA-sequencing data

Kallisto (v.0.50.1) was used to extract transcript-level counts from the fastq files. The human transcriptome index constructed from the Ensembl reference transcriptome version 108 was used for quantification (https://github.com/pachterlab/kallisto-transcriptome-indices/releases). The tximport R package (v.1.32.0) was used to summarize transcript-level counts to gene-level and create a count matrix.

### Differential expression analysis (DEA) for PDTO bulk RNA sequencing data

The DESeq2 R package (v1.40.2) [16] was used for DEA. Prefiltering was performed to exclude features with fewer than five normalized counts across n samples, where n equaled the lowest number of samples in the groups compared. Sequencing batch was included as a covariate in the design formula. The biomaRt R package (v.2.60.0) [17] was used to convert Ensembl gene IDs to Entrez gene IDs and symbols. The EnhancedVolcano R package (v.1.22.0) [18] was used to create volcano plots to visualize the DEA results.

### Pathway activity inference from PDTO bulk RNA-seq data

R implementation of the decoupleR framework (v.1.5.0) [19] was used to infer pathway activities from PDTO bulk RNA-seq data. The PROGENy [20] weights of the top 500 features were retrieved, and the stat values from the DEA results (DESeq2 output) were used as inputs for the multivariate linear model.

### Gene set enrichment analysis

For each DEA comparison, gene set enrichment analysis (GSEA) was performed with the fgsea [21] method implemented in the clusterProfiler R package (v.4.12.0) [22], using the curated (C2) and hallmark (H) gene sets available in the Human Molecular Signatures database (MSigDb) [23]. Pathways with Benjamini-Hochberg adjusted *p* values < 0.05 were considered significantly enriched.

### Molecular subtypes of PDTOs

The raw count matrix was filtered to exclude features with zero counts across all samples and variance stabilizing transformation was applied with DESeq2::vst() function with “blind = FALSE” setting. IMF classification was implemented using the CMScaller package [24], using the transformed count matrix as input and the gene sets published by Joanito et al. [25] as the template. The gene sets “iCMS2_Up” and “iCMS3_Down” were combined to form the iCMS2 class, whereas “iCMS2_Down” and “iCMS3_Up” were combined to form the iCMS3 class. PDTO samples were classified as iCMS2 or iCMS3 if the distance between a PDTO sample and the corresponding template was lower than 1000 random permutations of the gene labels (FDR < 0.05). Samples with FDR values higher than the cut-off were defined as “Undefined”.

### Reanalysis of the CNP0004138 scRNA-seq dataset

Preprocessed scRNA-seq data from the CNP0004138 project were downloaded from the China National GeneBank Database [26]. The celltypist [27] Python package (v.0.1.9) was used to annotate cell types using the “Immune_All_Low” model. Cells annotated as “Epithelial cells” were retained for downstream analyses. For each treatment stage (pre- and post-treatment), Seurat’s FindMarkers function was used to identify differentially expressed genes between the clinical response groups. Differentially expressed genes overlapping with ISGs were visualized on a lollipop plot.

EPSTI1^+^ epithelial cells were defined as cells with non-zero normalized *EPSTI1* expression. Then, for each sample, the percentage of EPSTI1^+^ cells relative to all epithelial cells was calculated and stacked bar plots were used to compare EPSTI1^+^ cell abundances across clinical response groups. To infer pathway activities in epithelial cells, R implementation of the decoupleR framework (v.1.5.0) [19] was used. The PROGENy [20] weights of the top 500 features were retrieved, and normalized gene expression matrix was used as input for the multivariate linear model. Inferred pathway activities were scaled and subsequently used for visualization.

### Bulk RNA-seq deconvolution

We applied InstaPrism [28] to infer cell type-specific transcriptome profiles from the GSE209746 and GSE50760 datasets. As a single-cell reference, we used the precompiled CRC_refPhi derived from Pelka et al. [29], which comprises 15 cell types and 98 cell states.

Raw read counts were used as input for deconvolution and epithelial tumor (EpiT)-specific read counts were extracted from the deconvolution output. Variance-stabilizing transformation (vst) was performed with DESeq2. In the GSE50760 dataset, normalized *EPSTI1* expression was compared between patient-matched normal colon tissue vs primary tumor and primary tumor vs metastatic tumor pairs. In the GSE209746 dataset, normalized *EPSTI1* expression was

compared between the clinical response groups (pCR vs pPR).

### *EPSTI1* knockdown and overexpression experiments

Caco-2, HCT116, and SW-48 KRAS G12V/+ cells were gifted by Ari Ristimäki’s research group (University of Helsinki). HCT116 and SW48 KRAS G12V/+ cells were grown in RPMI 1640 medium supplemented with 10% fetal bovine serum, 1% GlutaMAX, and 1% penicillin-streptomycin solution. Caco-2 cells were grown in DMEM supplemented with 10% fetal bovine serum, 1% GlutaMAX, and 1% penicillin-streptomycin solution. The Genomics Unit of Technology Center, Institute for Molecular Medicine Finland (FIMM) authenticated these cell lines using the GenePrint24 system (Promega).

RNA interference and plasmid overexpression were used to modulate *EPSTI1* expression. Briefly, 3 × 10^5^ cells were plated in a 6-well plate, and on the next day, the cells were transfected with Negative Control siRNA, EPSTI1 siRNA (#4392420, s41293, Ambion), or EPSTI1 ORF (#RG217229, OriGene). Lipofectamine RNAiMAX reagent (#13778030, Invitrogen) was used for siRNA transfections, whereas polyethylenimine (PEI) was used for plasmid transfection.

To assess how *EPSTI1* knockdown and overexpression modulates drug response, cells were dissociated with TrypLE reagent (#12605028, Gibco) 72 h after transfection, and 3 × 10^3^ cells were replated in a 96-well plate in four technical replicates. On the next day, cells were treated with fluorouracil or irinotecan. Three days after drug treatment, alamarBlue cell viability reagent (A50100, Invitrogen) was used to measure cell viability. Cell viability data from were analyzed using a two-way linear model with *EPSTI1* knockdown (control siRNA vs EPSTI1 siRNA) and drug treatment (DMSO, fluorouracil, irinotecan) as fixed factors, including their interaction term (viability ~ group × treatment). Interaction *p*-values were used to assess whether *EPSTI1* knockdown altered drug sensitivity. Linear modeling was performed using the *lm()* function in R.

### Reverse-transcription quantitative PCR (RT-qPCR)

RT-qPCR was performed to validate *EPSTI1* knockdown and overexpression, as well as ISG expression profiling after drug treatments. In each experiment, total RNA was isolated using NucleoSpin RNA Plus kit (MN) and eluted in nuclease-free water. Equal amounts of total RNA (1000 ng) were reverse-transcribed using High-Capacity cDNA Reverse Transcription Kit (#4368814, Applied Biosystems) following manufacturer’s protocol. Reverse-transcription reactions were performed on a T100 Thermal Cycler (Bio-Rad) or Veriti Thermal Cycler (Applied Biosystems). Quantitative PCRs were performed using PowerTrack SYBR Green Master Mix (A46109, Applied Biosystems), using 20 ng cDNA input per reaction and three technical replicates. *RPS13* was used to normalize gene expressions. Delta Ct values were calculated by subtracting the Ct value of target genes from that of *RPS13*, which are directly proportional to gene expression. Primer sequences are shown in Supplementary Table 3.

### Validating chemotherapy-induced ISG expression at protein level

Flow cytometry was used to analyze chemotherapy-induced changes in EPSTI1 protein expression. HCT116 cells were treated with DMSO (solvent control), fluorouracil (1 µM) or irinotecan (1 µM) for 72 h. IFN-gamma (10 ng/ml) was used as a positive control to induce EPSTI1 expression. Cells were fixed with 2% PFA solution for 1 h at RT, blocked with 1X PBS + 2% BSA solution for 15 min at RT, stained with the EPSTI1 antibody (Proteintech, 11627-I-AP, lot: 10619, dilution 1:500) for 30 min at RT, and stained with the Alexa Fluor 488-conjugated goat anti-rabbit IgG secondary antibody (Invitrogen, A-11008, dilution 1:500) for 30 min at RT. Flow cytometry was performed on a CytoFlex instrument and 10,000 events were recorded for each sample. Unstained cells and cells stained with the secondary antibody were used to check background staining. Median fluorescence intensity (MFI) was used to quantify and compare EPSTI1 expression.

For the WB experiment, HCT116 cells were treated with DMSO (0.1% solution, negative control), IFN-gamma (10 ng/ml, positive control), fluorouracil (1 µM), and irinotecan (1 µM). At indicated time points (1, 24, 48, and 72 h), cells were lysed in RIPA buffer supplemented with protease-phosphatase inhibitors (Thermo Scientific, 78440) and incubated on ice for 30 min. Cell lysates were clarified by centrifugation, and supernatant protein concentrations were measured by BCA assay (Thermo Scientific, A65453). Before SDS-PAGE, sample protein concentrations were normalized using RIPA buffer. Sample proteins were resolved on 8–16% SDS-PAGE gels (BioRad, #4568106) with total protein loading of 15 µg per sample. Proteins were transferred to nitrocellulose membranes using semi-dry transfer method (BioRad TransBlot Turbo). Following transfer, membranes were rinsed with TBS and blocked using EveryBlot reagent (BioRad, #12010020). Membranes were incubated with primary antibodies for pSTAT1 (Invitrogen, #33-3400, dilution 1:1000), STAT1 (Cell Signaling Technology #9172, dilution 1:1000) and β-actin (R&D Systems, #MAB8929, 1:2000). For detection, a mixture of goat anti-mouse DyLight800 (Invitrogen, #35521, dilution 1:5000) and goat anti-mouse DyLight680 (Invitrogen, WF328091, dilution 1:5000) was utilized. Between antibody incubations, membranes were washed 3 × 10 min using TBST. Blots were imaged using Odyssey CLx (LI-COR) fluorescence scanner and image densitometry was performed using Image Studio Lite software (LI-COR). Gel and blot total protein loading (BioRad StainFree) were imaged on a Chemidoc MP imaging system (Bio-Rad).

### Effect of *EPSTI1* knockdown on apoptosis

To determine whether *EPSTI1* knockdown induced apoptotic cell death, HCT116 cells were transfected overnight with a negative control siRNA or *EPSTI1* siRNA (see section “*EPSTI1* knockdown and overexpression experiments”). On the next day, transfection mixture was replaced with normal growth medium or growth medium supplemented with 50 µM pan-caspase inhibitor Z-VAD-FMK (Selleckchem, S7023). Seventy-two hours after transfection, cells and supernatants were collected and stained with Pacific Blue™ Annexin V (BioLegend, 5640918) and propidium iodide according to the manufacturer’s instructions and immediately analyzed on a CytoFlex flow cytometer.

Caspase activity was assessed using Caspase-3/7, Caspase-8 and Caspase-9 Multiplex Activity Assay Kit (abcam, ab219915), which measures the cleavage of target‑specific substrates for caspase‑3/7, caspase‑8, and caspase‑9. Briefly, 3 × 10^3^ HCT116 cells were plated in a 96-well plate in four replicates and cells were transfected overnight with a negative control siRNA or *EPSTI1* siRNA (see section “*EPSTI1* knockdown and overexpression experiments”). On the next day, transfection mixture was replaced with normal growth medium or growth medium supplemented with 50 µM pan-caspase inhibitor Z-VAD-FMK (Selleckchem, S7023). Seventy-two hours after transfection, cells were incubated with the corresponding caspase substrate reagent, and fluorescence intensity for each caspase was measured from the same wells on a Tecan Spark multimode microplate reader. For each caspase target, blank wells containing assay reagent without cells were included. The mean blank signal was calculated separately for each caspase, and sample fluorescence values were blank‑corrected by subtracting the target‑specific mean blank intensity.

### Immunohistochemistry

Paired biopsy and resection FFPE tissue samples from 15 patients were retrieved from pathology archives and reviewed by a pathologist. Biopsy and resection tissues for each patient were cut onto the same SuperFrost Plus slide at 4 µm thickness at Helsinki University Hospital. Chromogenic immunohistochemistry was performed with 3,3’-Diaminobenzidine (DAB) using Autostainer 480S automated IHC stainer (Epredia, Part No: A80500485) at Tampere University. Candidate antibodies and suitable dilutions were optimized using test tissues consisting of normal human appendix, tonsil and rectum tissues. Appropriate staining patterns were visually confirmed by a pathologist (JV). Single-plex immunohistochemical staining was done in several batches using EPST1 polyclonal antibody (Proteintech, 11627-I-AP, lot: 10619, dilution 1:400), CK20 (clone SP33, abcam, ab64090, lot: 1038252-5, dilution 1:200), CD3 (clone PS1, Neomarkers, MS-401-S1, lot: 401S801L, dilution 1:200), CD8 (clone C8/144B, Dako, M7103, lot: 20066516, dilution 1:200) and CD45 (clone D9M8I, CellTechnologies, 13917, lot: 10, dilution 1:200). Slides were baked for 30 min in 62 °C and dewaxed using Autostainer XL (Leica), followed by heat-induced antigen retrieval with Tris-EDTA-based buffer, pH 9.0 (10 mM (0.01 M) Tris-Base, 1 mM (0.001 M) EDTA Solution, 0.05% Tween 20) at 102 °C for 3 min (Retriever 2100, Aptum Biologics Ltd). Primary and secondary antibodies were incubated for 30 min at room temperature.

The slides were digitized with a 20× objective magnification using NanoZoomer S60 slide scanner (Hamamatsu Photonics, Hamamatsu City, Japan, resolution 0.4417 µm/pixel).

### Image analysis for immunohistochemistry

The digitized images of immunohistochemistry slides were processed using QuPath (v.6.0) [30]. Only representative tissue areas with successful staining were included for further analyses, while regions with tissue folds, inadequate staining or out of focus areas were excluded. Tissue compartments were manually annotated and classified into epithelial, stromal, tumor center (CT) and invasive margin (IM) areas. A multi-polygon ROI approach was used, where a single composite annotation per compartment was created for each slide by combining multiple representative sub-regions into one annotation object. Detection accuracy was visually validated by a pathologist (JV).

Regions of interest for EPSTI1 and CK20-stained biopsy and resection samples encompassed epithelial and stromal compartments. CK20 was restricted to epithelial regions since the marker is not expressed in stroma. Both the percentage of tumor cells stained for EPSTI1, and the staining intensity were used in analyses, while CK20 expression was quantified using staining intensity only.

For CD3, CD8 and CD45-stained samples, representative areas from the tumor center and the invasive margin from biopsy and resection tissues were selected for analysis. The invasive margin was defined as a region extending approximately 500 µm into the tumor and 500 µm into the normal tissue from the visible tissue frontier. In cases where insufficient tumor was present to accommodate this width, invasive margin was defined using equal proportional distances (50%/50%) on each side of the tumor-normal boundary.

The built-in cell detection function was used to detect cell staining intensities for EPSTI1 and CK20, and built-in positive cell count function was used to detect immune cells within compartments of interest. Immune cells were recognized by CD3, CD8 or CD45 expression and tumor cells were identified through morphology and CK20 staining.

### Statistical testing and data visualization

Statistical analyses and data visualization were performed using R version 4.4.1. The Wilcoxon rank-sum test was used to compare drug response estimates (AUC values) and gene expression between the two independent groups and Wilcoxon signed-rank test was used to compare gene expression between paired samples. The threshold for statistical significance was set to 0.05. Where appropriate, *p* values were adjusted with Benjamini-Hochberg or Bonferroni procedures.

Heatmaps were created using the ComplexHeatmap (v.2.24.1) package [31, 32] (RRID:SCR_017270). Seurat (v.5.0.0) (RRID:SCR_007322) and scCustomize (v.3.2.4) (RRID:SCR_024675) packages were used to visualize scRNA-seq data. Other plots were created using the ggpubr (v.0.6.2) package (RRID:SCR_021139).

Drug testing on PDTOs was performed once as an exploratory analysis due to the resources required for parallel drug testing and bulk RNA-seq, and to mitigate altered drug sensitivity profiles over prolonged culture time. Flow cytometry experiments to detect EPSTI1 protein expression and apoptosis were performed three times using independent biological replicates. For cell-based assays, biological replicates represent independent cell cultures initiated on different days.

The associations of EPSTI1 expression between patient-paired biopsy and resection specimens within epithelial and stromal compartments were evaluated with Wilcoxon signed-rank test. All reported *p* values for correlation analyses are BH-adjusted unless otherwise stated.

## Results

### CRC PDTOs as in vitro models to study epithelial cell-specific drug responses

To investigate epithelial cancer cell-specific responses to standard-of-care chemotherapeutics in CRC (fluoruracil, irinotecan, and oxaliplatin), we analyzed a subcohort of PDTOs established in the iCAN-PANORG project (Pan-cancer organoid biobank for precision medicine in abdominal cancers) and the SYNCOPE (Systemic Neoadjuvant and adjuvant Control by Precision Medicine in Rectal Cancer) trial (ClinicalTrials.gov ID NCT04842006). PDTOs were established from tumor specimens of 75 patients with colon or rectal adenocarcinoma (“Methods”; Table 1). To validate PDTO models, we performed whole exome sequencing (WES) on representative PDTOs (*n* = 44), which confirmed that canonical CRC driver mutations including *APC*, *TP53*, *KRAS*, and *BRAF* (Supplementary Fig. 1A).

**Table 1:** Overview of data modalities and patient characteristics.

		Patient characteristics								
Patient ID	PDTO ID	Age	Sex	Anatomical site	MMR status	Pretreatment	Pretreatment type	Radiological TNM	Pathological TNM	Final stage
CRC561	CRC561ORG	65	Female	Rectum	MMRp	No		rT1/2N1aM0	pT2N1b	III
CRC997	CRC997ORG	72	Male	Rectum	MMRp	No		rT2N0M0	pT2N0	I
CRC321	CRC321ORG	61	Female	Rectum	MMRp	No		rT2N1M0	pT2N0	I
CRC153	CRC153ORG	52	Female	Rectum	MMRp	No		rT4aN0M0	pT3N1c	III
CRC074	CRC074ORG	45	Female	Rectum	MMRp	No		rT1/2N0M0	pT1N0	I
CRC228	CRC228ORG	80	Male	Rectum	MMRp	No		rT3bN0M0	pT2N2a	III
CRC146	CRC146ORG	73	Female	Colon	MMRd	No		rT4N2M0	pT4bN0	II
CRC634	CRC634ORG	70	Male	Rectum	MMRp	Yes	RT	rT4aN0M0	ypT3N0	II
CRC049	CRC049ORG	69	Female	Rectum	MMRp	Yes	CRT	rT4bN1M0	ypT4bN0	II
CRC128	CRC128ORG	74	Male	Rectum	MMRp	No		rT3bN0M0	pT2N1b	III
CRC303	CRC303ORG	71	Female	Rectum	MMRp	No		rT3N0M0	pT2N0	I
CRC024	CRC024ORG	71	Female	Rectum	MMRp	No		rT3bN1M0	pT3N0	II
CRC839	CRC839ORG	72	Male	Rectum	MMRp	No		rT4aN1M0	pT4aN2b	III
CRC356	CRC356ORG_1	75	Male	Rectum	MMRp	No		rT1/2N0M0	pT2N0	I
CRC356	CRC356ORG_2	75	Male	Rectum	MMRp	No		rT1/2N0M0	pT2N0	I
CRC165	CRC165ORG	80	Male	Rectum	MMRp	Yes	CRT	rT3N2M0	ypT3N1a	III
CRC622	CRC622ORG	46	Female	Rectum	MMRp	No		rT3N1M0	pT2N0	I
CRC532	CRC532ORG_1	77	Male	Rectum	MMRp	Yes	CRT	rT3dN2M0	ypT3N2b	III
CRC532	CRC532ORG_2	77	Male	Rectum	MMRp	Yes	CRT	rT3dN2M0	ypT3N2b	III
CRC882	CRC882ORG	45	Female	Rectum	MMRp	Yes	CRT	rT4bN2M0	ypT3N0	II
CRC879	CRC879ORG	67	Male	Rectum	MMRp	No		rT3N1M1	ypT3N0	IV
CRC899	CRC899ORG	58	Male	Rectum	MMRp	Yes	CRT	rT3bN1M0	ypT2N1c	III
CRC297	CRC297ORG	73	Male	Rectum	MMRp	No		rT3aN1M0	pT3N0	II
CRC986	CRC986ORG	61	Male	Rectum	MMRp	No		rT4aN0M1	pT3N2a	IV
CRC283	CRC283ORG	57	Male	Rectum	MMRp	Yes	CRT	rT3N2M0	ypT0N0	0
CRC932	CRC932ORG_1	36	Male	Colon	MMRp	Yes	CRT	rT3N2M1	no resection	IV
CRC621	CRC621ORG	75	Female	Rectum	MMRp	No		rT3aN1M0	pT2N0	I
CRC517	CRC517ORG	51	Male	Rectum	MMRp	No		rT1/2N0M0	pT2N1b	III
CRC212	CRC212ORG	70	Male	Rectum	MMRp	No		rT3bN0M0	pT2N0	I
CRC930	CRC930ORG_2	79	Female	Colon	MMRp	No		rT1/2N0M0	pT3N0	I
CRC860	CRC860ORG	76	Female	Rectum	MMRp	No		rT3bN1M0	pT3N1a	III
CRC872	CRC872ORG	77	Male	Rectum	MMRp	Yes	RT	rT3dN0M0	ypT3N0	II
CRC259	CRC259ORG	42	Male	Colon	MMRd	No		no imaging	pT1N0	I
CRC314	CRC314ORG	68	Female	Rectum	MMRp	No		rT2N0M0	pT2N0	I
CRC481	CRC481ORG	73	Male	Rectum	MMRp	No		rT2N1M0	pT3N0	II
CRC298	CRC298ORG	69	Female	Rectum	MMRp	Yes	CRT	rT4bN1M0	ypT3N0	II
CRC989	CRC989ORG	44	Male	Rectum	MMRp	Yes	CRT	rT4bN0	ypT0N0	0
CRC158	CRC158ORG	79	Male	Rectum	MMRp	No		rT3bN1M0	pT3N0	II
CRC299	CRC299ORG	78	Male	Rectum	MMRp	No		rT2N0M0	pT3N0	II
CRC911	CRC911ORG	41	Male	Rectum	MMRp	Yes	CRT	rT4bN1M0	ypT2N0	I
CRC406	CRC406ORG	84	Male	Rectum	MMRp	No		rT4aN1M1	pT3N0	IV
CRC967	CRC967ORG	77	Female	Rectum	MMRp	No		rT2N1M0	pT3N2b	III
CRC648	CRC648ORG	70	Male	Rectum	MMRp	No		rT3bN1M0	pT2N0	I
CRC292	CRC292ORG	69	Female	Rectum	MMRp	No		rT3bN1M0	pT3N1b	III
CRC836	CRC836ORG	65	Female	Rectum	MMRp	Yes	CRT	rT4N0M0	no resection	II
CRC994	CRC994ORG	86	Female	Colon	MMRd	No		rT1/2N0M1	pT3N2a	IV
CRC974	CRC974ORG	66	Male	Rectum	MMRp	No		rT4aN0M0	pT3N0	II
CRC348	CRC348ORG	73	Female	Rectum	MMRp	No		rT1/2N1M0	pT3N2a	III
CRC197	CRC197ORG	76	Male	Rectum	MMRp	No		rT3N1M0	pT3N1b	III
CRC516	CRC516ORG	93	Male	Rectum	MMRp	No		rT2N0M0	pT3N1a	III
CRC226	CRC226ORG	68	Male	Rectum	MMRp	No		rT3N1M0	pT2N0	I
CRC504	CRC504ORG	49	Female	Rectum	MMRp	No		rT4aN1M0	pT3N1a	III
CRC946	CRC946ORG	64	Female	Rectum	MMRp	No		rT1/2N1M1	pT2N1a	IV
CRC355	CRC355ORG	81	Male	Rectum	MMRp	Yes	CRT	rT4bN1M0	ypT1N0	I
CRC727	CRC727ORG	58	Female	Rectum	MMRp	Yes	RT	rT4bN1M0	ypT3N0	II
CRC945	CRC945ORG	82	Male	Rectum	MMRp	No		rT2N0M0	pT3N0	II
CRC245	CRC245ORG	71	Male	Rectum	MMRp	Yes	CRT	rT3cN2M0	no resection	III
CRC922	CRC922ORG	48	Male	Rectum	MMRp	Yes	RT	rT3N2M0	ypT2N0	I
CRC626	CRC626ORG	75	Male	Rectum	MMRp	Yes	CRT	rT3N1M0	ypT0N0	0
CRC262	CRC262ORG	62	Female	Colon	MMRp	No		rT3N1M0	pT4bN1bM1	IV
CRC390	CRC390ORG	52	Female	Colon	MMRp	No		rT1/2N0M0	pT2N0	I
CRC130	CRC130ORG	60	Male	Colon	MMRp	No		rT3/4N1M0	pT3N1b	III
CRC372	CRC372ORG	69	Male	Colon	MMRp	No		rT2N1M0	pT3N0	II
CRC003	CRC003ORG	63	Female	Colon	MMRp	No		rT1/2N0M0	pT3N0	II
CRC374	CRC374ORG	68	Male	Colon	MMRp	No		rT3N1M0	pT3N1b	III
CRC405	CRC405ORG	68	Male	Colon	MMRp	No		rT2N0M0	pT3N0	II
CRC770	CRC770ORG	76	Female	Colon	MMRp	No		rT3N2M0	pT3N2b	III
CRC870	CRC870ORG	72	Male	Colon	MMRd	No		rT3N1M0	pT3N1b	III
CRC698	CRC698ORG	70	Male	Colon	MMRp	No		rT1/2N1M0	pT3N0	II
CRC650	CRC650ORG	71	Male	Colon	MMRp	No		rT3N1M0	pT3N0	II
CRC040	CRC040ORG	55	Male	Colon	MMRp	No		rT2-3N1M0	pT3N2b	III
CRC973	CRC973ORG	68	Female	Colon	MMRd	No		rT1N2M0	pT2N1a	III
CRC033	CRC033ORG	79	Male	Colon	MMRp	No		rT1-2N0M0	pT3N1a	III
CRC103	CRC103ORG_1	42	Female	Rectum	MMRp	Yes	CRT	rT4bN2M0	ypT3N1bM1	IV
CRC103	CRC103ORG_2	42	Female	Rectum	MMRp	Yes	CRT	rT4bN2M0	ypT3N1bM1	IV
CRC109	CRC109ORG	50	Male	Rectum	MMRp	No		rT3dN1M1	no resection	IV
CRC329	CRC329ORG	34	Male	Rectum	MMRd	Yes	CRT	rT4N2M0	ypT4bN0	II
CRC669	CRC669ORG	60	Female	Rectum	MMRp	Yes	CRT	rT4bN2M0	ypT2N0	I
CRC076	CRC076ORG	69	Female	Rectum	MMRp	Yes	CRT	rT4bN0M1	ypT3N0	IV
CRC777	CRC777ORG	76	Male	Rectum	MMRp	Yes	CRT	rT3cN1M0	ypT4aN1bM1	IV
CRC491	CRC491ORG	66	Male	Rectum	MMRp	Yes	CRT	rT4N2M0	ypT2N0	I
CRC547	CRC547ORG	73	Male	Rectum	MMRp	Yes	CRT	rT4bN2M0	ypT0N0	0
CRC733	CRC733ORG	49	Male	Rectum	MMRp	No		rT2N0M0	pT2N0	I
CRC016	CRC016ORG	46	Male	Rectum	MMRp	Yes	RT	rT3N0M0	ypT0N0	0
CRC357	CRC357ORG	55	Female	Rectum	MMRp	Yes	RT	rT4bN2M0	ypT2N0	I
CRC732	CRC732ORG	62	Male	Rectum	MMRp	Yes	RT	rT4bN2M0	ypT0N0	0
CRC248	CRC248ORG	54	Female	Rectum	MMRp	No		local recurrence	no resection	NA
CRC325	CRC325ORG	37	Male	Rectum	MMRp	Yes	CRT	rT3N2M0	ypT3N1a	III

*MMRp* Mismatch repair proficient, *MMRd* Mismatch repair deficient, *RT* Radiotherapy, *CRT* Chemoradiotherapy.

**Fig. 1:**
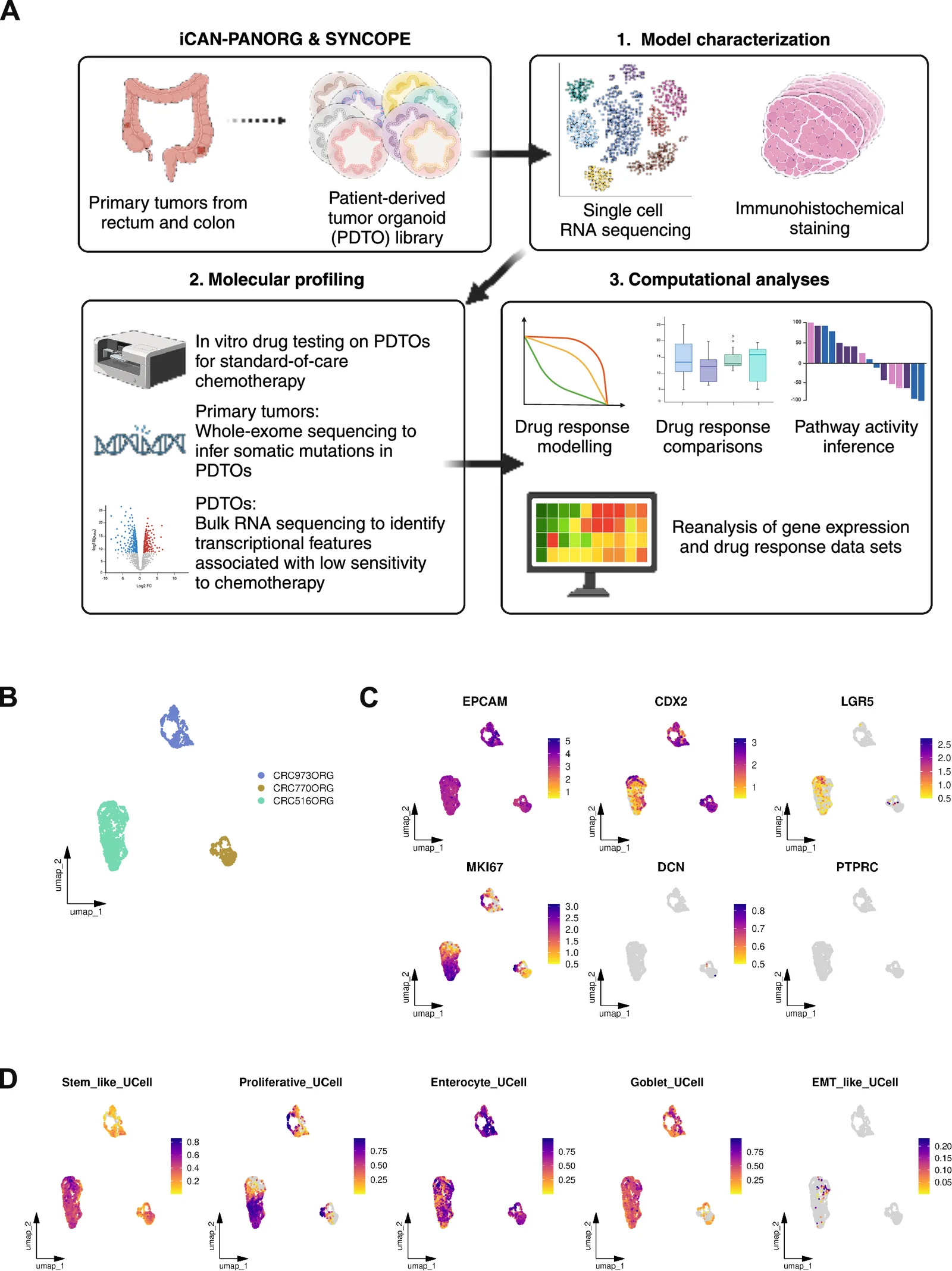
Studying epithelial cell-specific drug responses with CRC PDTOs. **A** Overview of the study design. **B** UMAP projections of the scRNA-seq data of three representative PDTOs, showing clustering by patient. **C** Feature plots showing strong expression of epithelial cell identity genes (*EPCAM* and *KRT20*), along with heterogeneous expression of stemness (*LGR5*) and proliferation (*MKI67*) genes. Stromal cell (*DCN*) and immune cell (*PTPRC*) markers show minimal expression, which is consistent with the epithelial nature of the PDTOs. **D** UMAP projections of module scores corresponding to CRC-related epithelial gene programs illustrate intra- and inter-tumor heterogeneity.

To characterize the cellular composition of PDTOs further, we performed single-cell RNA sequencing (scRNA-seq) on three representative PDTOs. This revealed pronounced inter-tumor heterogeneity with clusters segregating by patient of origin (Fig. 1B). The expression of canonical epithelial markers (*EPCAM*, *CDH1*, and *KRT20*) and intestinal lineage marker (*CDX2*) confirmed epithelial tumor identity, whereas the absence of stromal (*VIM*, *DCN*, *COL1A2*) and immune (*PTPRC*, *CD3D*) markers confirmed the absence of non-epithelial cell types. Cell subpopulations within the epithelial compartment expressed stem/progenitor markers (*LGR5*, *SOX9*, *PROM1*) and proliferation markers (*MKI67*, *PCNA*), indicating the presence of tumor-initiating and highly cycling populations (Fig. 1C, Supplementary Fig. 1B). We calculated gene module scores for previously defined CRC-related cell types and states, which highlighted distinct stem-like, proliferative, enterocyte-like, goblet-like, and EMT-like programs (Fig. 1D). Together, these results demonstrate that CRC PDTOs recapitulate the epithelial heterogeneity of primary tumors but lack stromal and immune components.

**Fig. 2:**
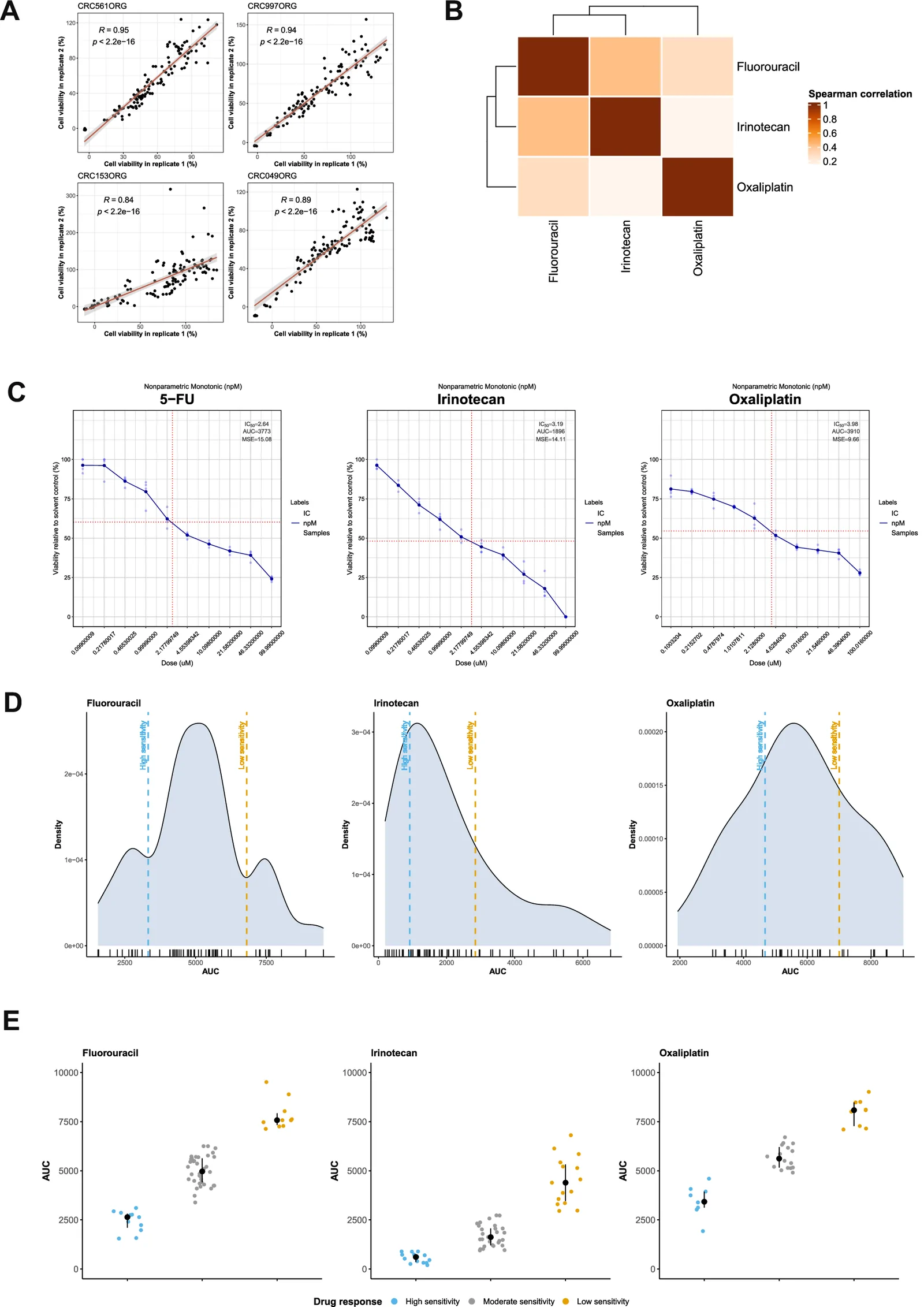
Drug response modeling and classification. **A** In vitro drug responses of PDTOs are reproducible across passages. **B** In vitro drug responses to standard-of-care chemotherapeutics did not show strong correlations. **C** Representative dose-response curves for fluorouracil, irinotecan, and oxaliplatin. **D** The distributions of AUC values are distinct for each chemotherapeutic agent. The dashed lines indicate classification thresholds. **E** Dot plots showing AUC values with respect to in vitro drug response classification groups.

We next assessed in vitro drug sensitivity of 75 PDTOs to fluorouracil, irinotecan, and oxaliplatin. PDTOs were treated with chemotherapeutics for 5 days, and dose-response curves were generated to calculate the point estimates of drug responses (AUC values). After quality control (“Methods”), 62 PDTOs met criteria for inclusion. Technical replicates across two timepoints showed high reproducibility (Spearman’s r 0.84-0.95), validating assay robustness (Fig. 2A). In vitro drug responses of PDTOs to chemotherapeutics did not show strong correlations (Fig. 2B), consistent with non-overlapping mechanisms of action. Representative dose-response curves are shown in Fig. 2C. PDTOs displayed heterogeneous in vitro drug responses to chemotherapeutics, with irinotecan showing the highest efficacy (Fig. 2D). Using the identified AUC thresholds (see “Methods”), we classified the drug responses of PDTOs into low, moderate, and high chemosensitivity groups (Fig. 2E), with the intention of achieving an analogous response classification mimicking no clinical response, partial response, and complete response categories.

### iCMS2 molecular subtype is associated with lower chemosensitivity to fluorouracil

**Fig. 3:**
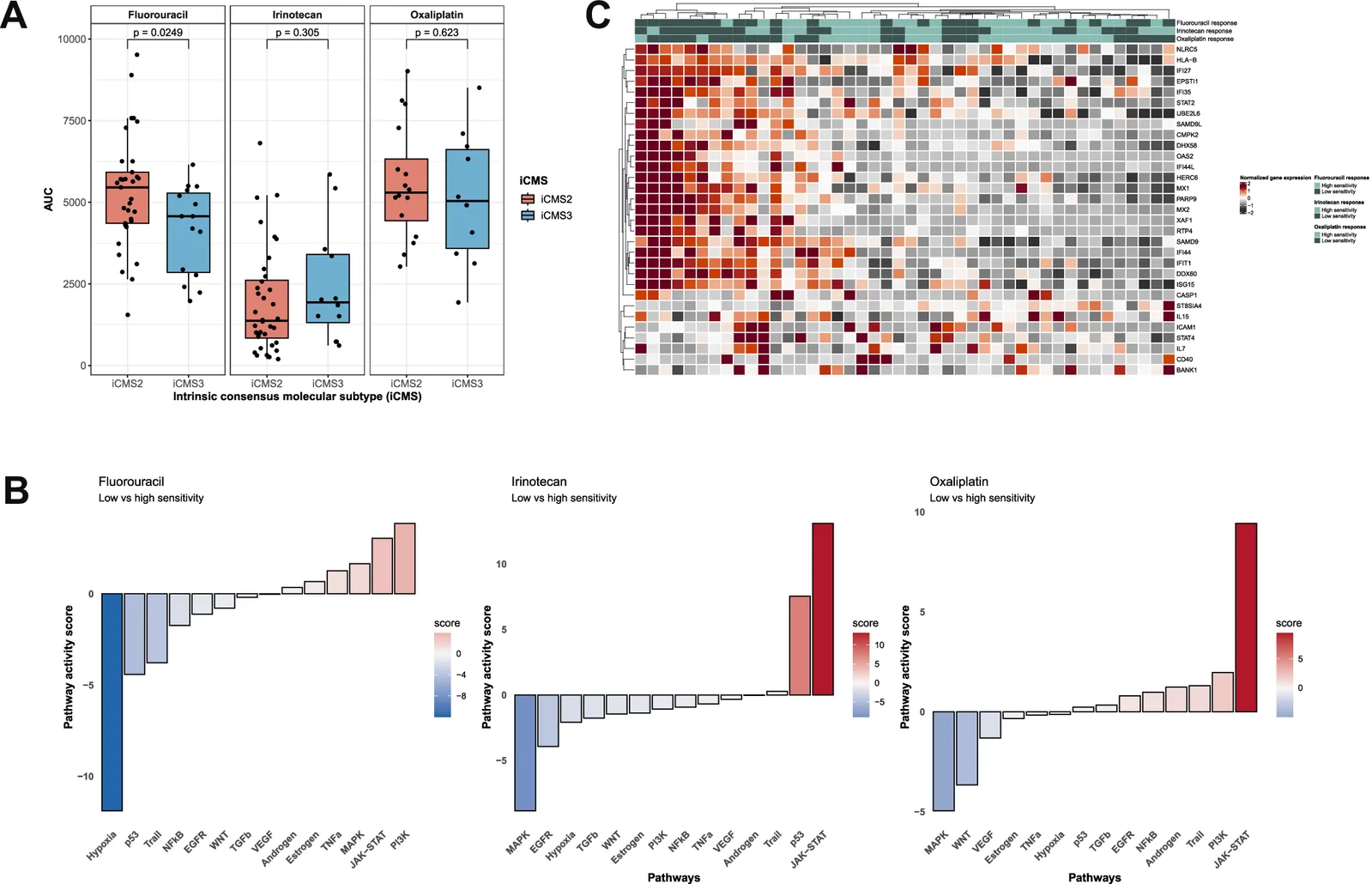
Linking transcriptomic features to in vitro drug responses. **A** iCMS2 PDTOs showed lower chemosensitivity to fluorouracil (*p* = 0.0249, Wilcoxon rank-sum test). **B** Waterfall plots showing differences in inferred pathway activities between PDTOs with low and high chemosensitivity to fluorouracil, irinotecan, and oxaliplatin. **C** Heatmap showing ISG expression across CRC GDSC cell lines (*n* = 44). Notably, a subset of ISGs have higher expression in CRC cell lines with low chemosensitivity to chemotherapy.

We next investigated whether the molecular subtypes of PDTOs are associated with in vitro drug responses. The consensus molecular subtype (CMS) classification [33] based on bulk-level gene expression profiles is not directly applicable to epithelial PDTOs; therefore, we used the epithelial cell-derived IMF classification system [25]. Using the CMScaller algorithm, we classified 36 PDTOs as iCMS2 and 15 as iCMS3 (Supplementary Table 1). Compared with iCMS PDTOs, iCMS2 PDTOs had a greater AUC for fluorouracil, indicating lower chemosensitivity (*p* = 0.0249, Wilcoxon rank-sum test) (Fig. 3A). No statistically significant differences were observed for irinotecan and oxaliplatin (irinotecan, *p* = 0.305; oxaliplatin, *p* = 0.623).

### High JAK-STAT pathway activity in CRC PDTOs is associated with lower chemosensitivity

To identify the transcriptomic features associated with poor in vitro drug responses, we performed differential expression analysis (DEA) between (1) PDTOs with low and high chemosensitivity and (2) PDTOs with moderate and high chemosensitivity (“Methods”) (Supplementary Fig. 2A). We then used multivariate linear modeling to infer pathway activities based on the differentially expressed genes between the groups.

The JAK-STAT pathway was ranked as the most active pathway in PDTOs with low chemosensitivity to irinotecan and oxaliplatin and the second most active pathway in PDTOs with low chemosensitivity to fluorouracil (Fig. 3B), due to high expression of ISGs, including *IFI6*, *IFIT1*, *IFIT2*, *IFIT3*, *ISG15*, and *MX1* (Supplementary Fig. 2B). In parallel, we performed gene set enrichment analysis (GSEA) for hallmark gene sets (H) and curated gene sets (C2) in MSigDB to identify transcriptional programs associated with low sensitivity to each chemotherapeutic. Consistent with high JAK-STAT pathway activity, hallmark gene sets for interferon alpha and interferon gamma responses were ranked among the top gene sets in PDTOs with low sensitivity to irinotecan and oxaliplatin (Supplementary Table 2).

The lack of public databases linking gene expression profiles of PDTOs to their drug sensitivity profiles prompted us to examine the Genomics of Drug Sensitivity in Cancer (GDSC) database [34, 35], which provides gene expression and drug sensitivity data of cell line models of CRC. Focusing on 44 CRC cell lines with available gene expression and drug sensitivity data, we used the median IC50 values as a threshold to classify cell lines with low or high sensitivity to fluorouracil, irinotecan, and oxaliplatin. Multiple ISGs, including *STAT1*, *IFI6*, *IFIT1*, *IFIT2*, and *IFIT3*, were highly expressed in cell lines with low sensitivity to chemotherapeutics (Fig. 3C), further supporting the association between ISG expression and the low chemosensitivity of epithelial cancer cells.

### Incomplete pathological response is associated with sustained epithelial ISG expression

To assess whether epithelial ISG expression is linked to clinical response, we reanalyzed a publicly available scRNA-seq dataset (CNP0004138) comprising rectal tumor tissues collected from patients before and after neoadjuvant chemotherapy without radiotherapy [26]. ISG expression across epithelial cells was generally sparse, with the exceptions of *IFI27* and *HLA-B* (Supplementary Fig. 3).

**Fig. 4:**
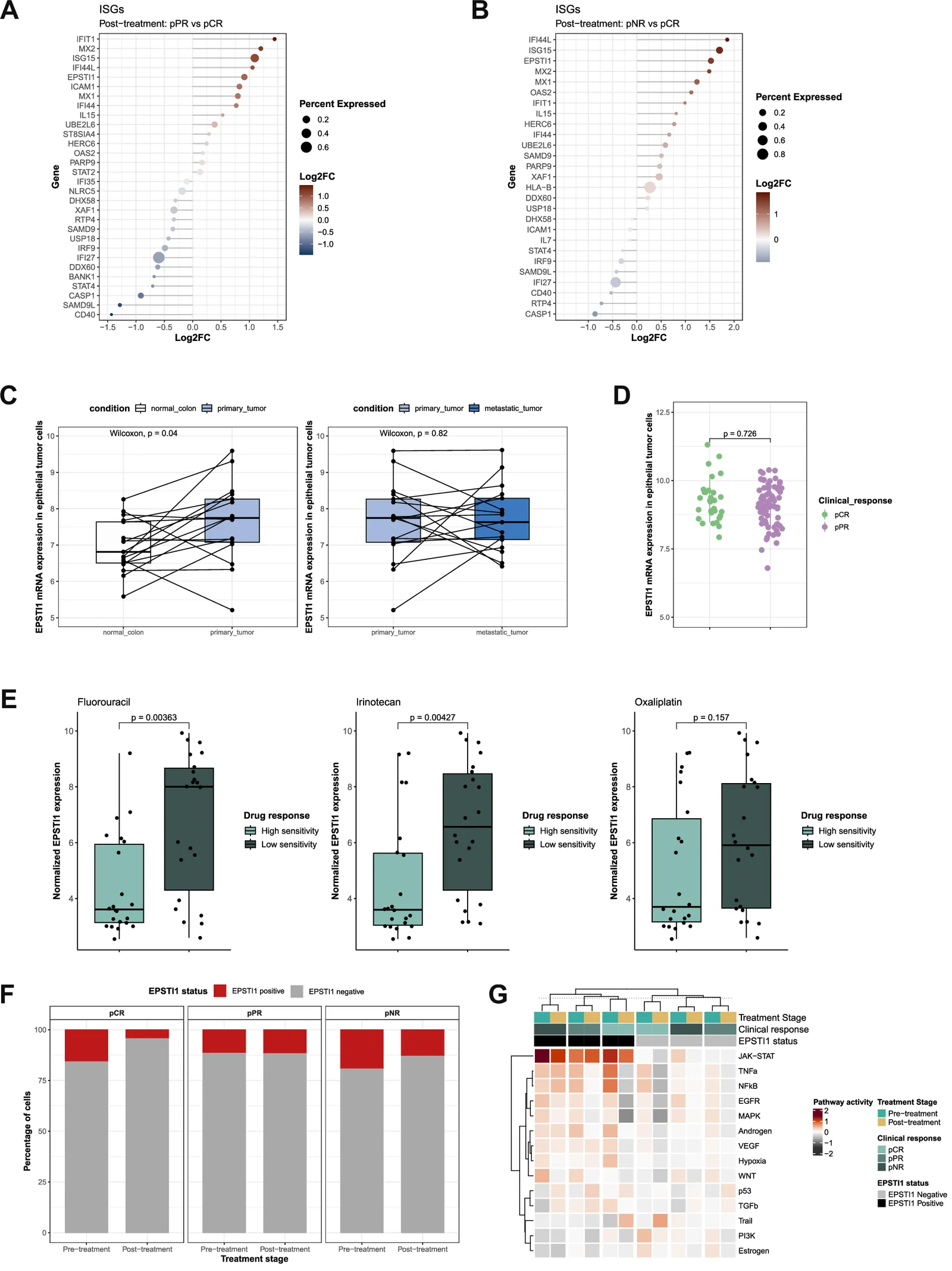
Chemotherapy-modulated ISG expression is linked to incomplete pathological response. **A**, **B** After chemotherapy, a subset of ISGs had higher expression in tumors with pPR and pNR. **C** Primary CRC tumors had higher *EPSTI1* expression compared to patient-matched normal colon tissues. Liver metastases, on the other hand, showed heterogeneous *EPSTI1* overexpression compared to patient-matched primary tumors. **D** *EPSTI1* expression in treatment-naïve primary tumors did not differ between clinical response groups. **E** *EPSTI1* expression was higher in CRC cell lines with low in vitro sensitivity to standard-of-care chemotherapeutics (Fluorouracil, *p* = 0.00363; Irinotecan, *p* = 0.00427; Oxaliplatin, *p* = 0.00193, Wilcoxon rank-sum test). **F** Chemotherapy led to a sharp reduction in the fraction of EPSTI1^+^ cells in pCR tumors, but not in pPR and pNR tumors. **G** Heatmap showing chemotherapy-induced changes in pathway activities with respect to EPSTI1 status and clinical response. pCR tumors have active Trail pathway, which distinguishes these tumors from pPR and pNR tumors.

We focused on post-treatment samples to capture chemotherapy-induced transcriptional changes and stratified tumors based on pathological response: complete response (pCR), partial response (pPR), or no response (pNR). Tumors with incomplete pathological response (pNR or pPR) showed higher expression of ISGs, including *IFIT1*, *MX1*, *ISG15*, and *EPSTI1*, compared to complete responders (pCR) (Fig. 4A, B). This finding suggests that sustained epithelial ISG expression persists in tumors with incomplete pathological responses and may reflect a dynamic transcriptional state enabling epithelial tumor cells to survive genotoxic stress induced by chemotherapy.

### *EPSTI1* expression is upregulated in primary CRC tumors and dynamically regulated in response to chemotherapy

Among the differentially expressed genes with higher expression in pNR and pPR tumors, we focused on epithelial-stromal interaction 1 (*EPSTI1*) due to its reported roles in EMT regulation [36], cell migration and invasion, and anchorage-independent growth [37]. We hypothesized that *EPSTI1* may support survival of epithelial tumor cells in the context of chemotherapy.

To investigate *EPSTI1* expression during CRC progression, we deconvoluted the bulk RNA-seq dataset GSE50760, which comprises patient-matched normal colon, primary tumor, and liver metastasis samples (17 patients, 51 samples) and extracted epithelial tumor cell-specific gene expression profiles. *EPSTI1* expression was higher in primary tumors compared to matched normal tissues (*p* = 0.04, Wilcoxon signed rank test). In contrast, metastatic tumors showed heterogeneous *EPSTI1* expression relative to matched primary tumors (*p* = 0.82, Wilcoxon signed rank test), indicating divergent regulation of *EPSTI1* expression during disease progression (Fig. 4C).

We next asked whether baseline *EPSTI1* expression in epithelial tumor cells correlates with clinical response to chemotherapy. Using the bulk RNA-seq dataset GSE209746 comprising treatment-naïve rectal adenocarcinomas, we compared *EPSTI1* expression in epithelial tumor cells between pCR and pPR tumors. *EPSTI1* expression in epithelial tumor cells was not significantly different between pCR and pPR tumors (*p* = 0.726, Wilcoxon rank-sum test), indicating that *EPSTI1* expression is not a robust predictor of clinical response in treatment-naïve tumors (Fig. 4D). However, when we compared *EPSTI1* expression between CRC cells in the GDSC dataset, we observed higher *EPSTI1* expression in CRC cell lines with low chemosensitivity to fluorouracil, irinotecan, and oxaliplatin (Fig. 4E).

To assess whether *EPSTI1* expression reflects broader ISG activation in CRC tumors, we generated an ISG gene signature from a curated panel of ISGs excluding *EPSTI1* and computed ISG scores in both GSE50760 and GSE209746 datasets. *EPSTI1* expression was significantly positively correlated with the ISG signature score in both datasets, indicating that its expression mirrors activation of a broader ISG program (Supplementary Fig. 2C).

To test how EPSTI1^+^ cells are regulated by chemotherapy, we focused on the scRNA-seq dataset CNP0004138 and compared EPSTI1^+^ cell abundance. While treatment-naïve tumors showed comparable EPSTI1^+^ cell abundances across clinical response groups, following chemotherapy, these cells were selectively depleted in tumors with pCR but persisted in tumors with incomplete response (Fig. 4F). To explore a potential mechanism for this selective depletion, we compared inferred pathway activities in EPSTI1^+^ and EPSTI1^-^ epithelial cells across clinical response groups, which revealed high activity of tumor necrosis factor (TNF)-related apoptosis-inducing ligand (TRAIL) pathway only in pCR tumors (Fig. 4G), raising the hypothesis that TRAIL-mediated apoptosis could contribute to the selective elimination of EPSTI1^+^ cells. Taken together, evidence from bulk RNA-seq and scRNA-seq studies supports a model in which *EPSTI1* emerges as an epithelial cell-specific modulator under the selective pressure of chemotherapy.

### STAT1 mediates chemotherapy-induced ISG activation

*STAT1* is well-recognized for its tumor-suppressive roles [38], whereas *STAT3* and *STAT5* are linked to inflammation and tumor progression [39, 40]. To clarify which STAT family members associate with the epithelial ISG program linked to low chemosensitivity, we calculated gene-gene correlations in the bulk RNA-seq datasets GSE209746 and GSE50760. *STAT1* showed strong positive correlations with ISGs including *EPSTI1*, whereas *STAT3* and *STAT5* displayed distinct correlation patterns (Supplementary Fig. 4), suggesting differential STAT family involvement in ISG activation.

**Fig. 5:**
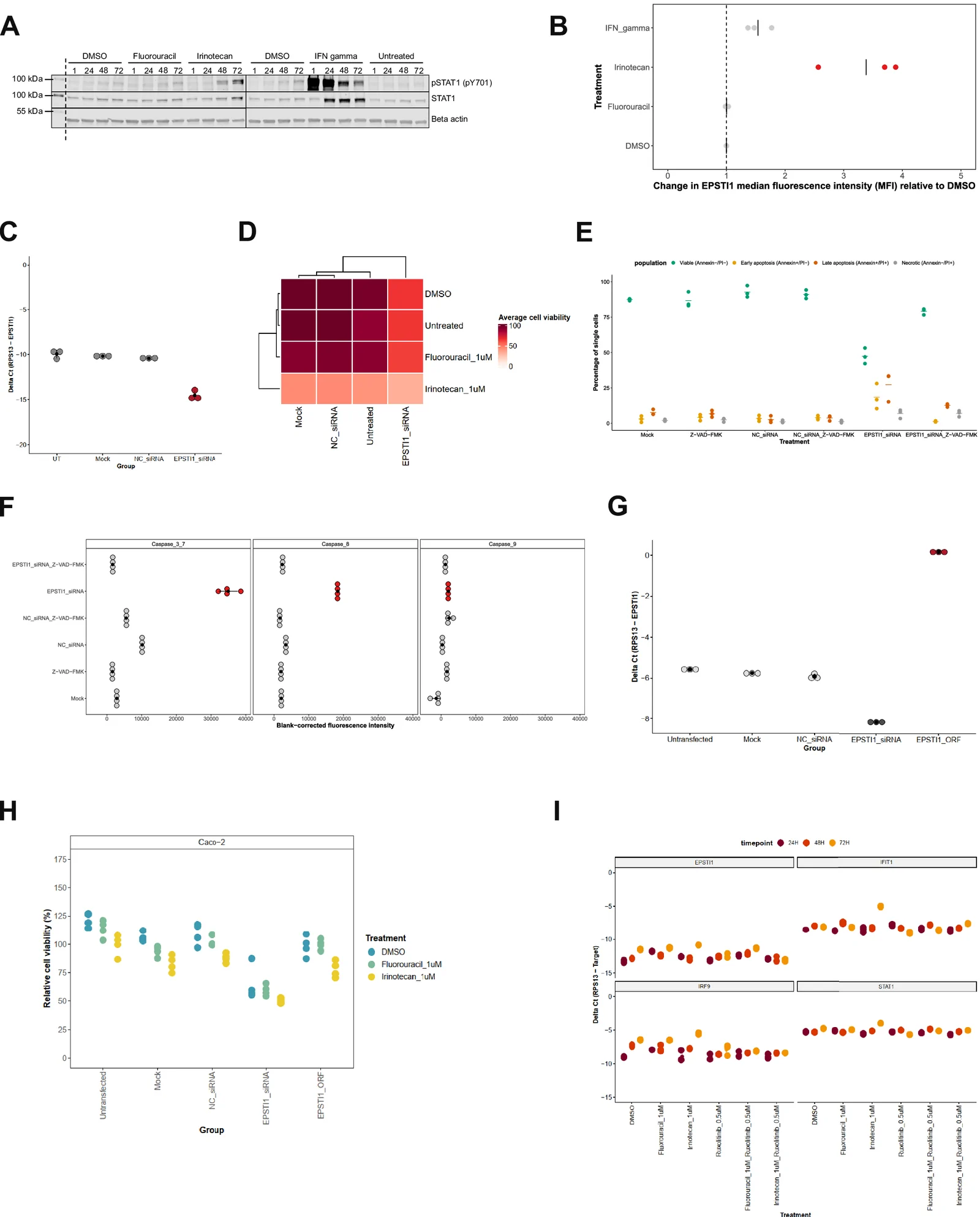
Functional roles of ISGs in chemosensitivity. **A** Temporal fluorouracil- and irinotecan-induced changes in STAT1 and pSTAT1 (Y701) expression in HCT116 cells. **B** Irinotecan-induced STAT1 activation leads to higher EPST1 expression. **C** RT-qPCR validation of *EPSTI1* knockdown in HCT116 cells. **D** *EPSTI1* knockdown reduced baseline cell viability and sensitized HCT116 cells to fluorouracil and irinotecan. **E**, **F** *EPSTI1* knockdown in HCT116 cells induces apoptotic cell death, which is partially restored with pan-caspase inhibitor Z-VAD-FMK. **G** *EPSTI1* knockdown in HCT116 cells induces apoptotic cell death via caspase-8 and caspase-3/7 activation. **G** RT-qPCR validation of *EPSTI1* knockdown (EPSTI1_siRNA) or overexpression (EPSTI1_ORF) in Caco-2 cells. **H** *EPSTI1* knockdown sensitized Caco-2 cells to fluorouracil and irinotecan, whereas overexpression reduced chemosensitivity. **I** Ruxolitinib counteracted chemotherapy-induced ISG expression by suppressing JAK-STAT pathway activity at the transcriptional level.

Next, we tested whether chemotherapy-induced ISG activation is translated to protein-level changes. To assess temporal dynamics of ISG activation, HCT116 cells were treated with fluorouracil (1 µM) or irinotecan (1 µM), and Western blotting was performed to check STAT1 and p-STAT1 (Y701) expression for up to 72 h. Treatment with fluorouracil or irinotecan for 1 h was insufficient to induce p-STAT1 expression, suggesting that these chemotherapeutics do not trigger an immediate, IFN-like STAT1 activation. At later time points (48 h and 72 h), irinotecan treatment led to a clear increase in total STAT1 and p-STAT1 (Y701) expression, whereas the effect of fluorouracil was weaker (Fig. 5A). Focusing on *EPSTI1* as the effector ISG, we also used flow cytometry to quantify changes in EPSTI1 protein expression after chemotherapy. Consistent with STAT1 activation results, irinotecan treatment led to a robust increase in EPSTI1 protein expression at 72 h, whereas the effect of fluorouracil was negligible (Fig. 5B). Collectively, these results confirm that chemotherapy-induced ISG activation is reflected at the protein level and further highlight mechanistic differences between fluorouracil and irinotecan in engaging the STAT1-EPSTI1 axis.

### *EPSTI1* supports CRC cell viability and modulates chemotherapy response

We next asked whether extrinsic IFN-gamma stimulation could induce ISG expression and reproduce the low-chemosensitivity phenotype. Short-term pretreatment of HCT116 cells with 1 ng/mL IFN-gamma induced ISG expression in a time-dependent manner (Supplementary Fig. 5A) but did not reduce chemosensitivity; instead, IFN-gamma-irinotecan combination was marginally more cytotoxic than irinotecan alone (Supplementary Fig. 5B). To test whether prolonged pretreatment might alter this effect, we pretreated HCT116 cells with 0.1 ng/mL IFN-gamma for 7 days. Despite clear induction of ISG expression (Supplementary Fig. 5C), prolonged pretreatment also failed to reduce chemosensitivity and remained marginally cytotoxic (Supplementary Fig. 5D). Thus, while extrinsic IFN-gamma stimulation robustly induces ISG expression, it does not recapitulate the stress-adaptive, low-chemosensitivity state observed in PDTOs. These results suggest that *EPSTI1* is a key contributor but not the sole driver of chemoresistance.

To test whether *EPSTI1* contributes to chemotherapy response, we used RNA interference to knock down *EPSTI1* expression in HCT116 cells. A two‑way linear model (viability ~ group × treatment) revealed a significant interaction between *EPSTI1* knockdown and irinotecan, but not fluorouracil, indicating that *EPSTI1* depletion sensitizes HCT116 cells to irinotecan under the experimental conditions. The negative but non‑significant interaction term for *EPSTI1* siRNA × fluorouracil is consistent with limited fluorouracil efficacy at 1 µM (Fig. 5C, D). Flow cytometry with Annexin V/PI staining showed a marked increase in apoptosis following siRNA-mediated *EPSTI1* knockdown in HCT116 cells. Compared with mock and NC siRNA controls, *EPSTI1* siRNA substantially reduced the viable cell population (Annexin^-^/PI^-^), and increased both early (Annexin^+^/PI^-^) and late apoptotic (Annexin^+^/PI^+^) fractions. Pan-caspase inhibitor Z-VAD-FMK partially reversed the apoptotic phenotype induced by *EPSTI1* knockdown (Supplementary Fig. 6; Fig. 5E). In line with these findings, treatment with *EPSTI1* siRNA induced caspase-3/7 activation, which was partially suppressed by caspase inhibitor Z-VAD-FMK., and induced caspase-8 activation, in line with previous observations in breast cancer (Fig. 5F). Together, these results indicate that loss of *EPSTI1* promotes caspase-8- and caspase-3/7-dependent apoptotic cell death, providing a mechanistic explanation of how *EPSTI1* supports cell fitness and modulates chemosensitivity.

To determine whether phenotypic changes induced by *EPSTI1* knockdown generalize beyond HCT116, we extended our analysis to Caco-2 and SW48 KRAS G12V/+ cell lines. In Caco-2 cells, *EPSTI1* knockdown reduced cell viability and enhanced chemosensitivity to fluorouracil and irinotecan, whereas *EPSTI1* overexpression reduced chemosensitivity (Fig. 5G, H). In SW48 KRAS G12V/+ cells, *EPSTI1* overexpression was confirmed with RT-qPCR but due to low baseline *EPSTI1* expression, knockdown could not be reliably assessed (Supplementary Fig. 5E). Nevertheless, phenotypic assays revealed enhanced chemosensitivity after *EPSTI1* knockdown and reduced chemosensitivity after *EPSTI1* overexpression, similar to the effects observed in HCT116 and Caco-2 cell lines (Supplementary Fig. 4F).

To assess how chemotherapy regulates EPSTI1 protein expression in the tumor microenvironment (TME) context, we also quantified EPSTI1 staining by IHC in patient‑matched pre‑treatment biopsies and post‑treatment resections taken after long pretherapy with capecitabine ± oxaliplatin. Both cytoplasmic EPSTI1 staining and the fraction of EPSTI1⁺ epithelial cells were reduced in post‑treatment samples, accompanied by a parallel decrease in stromal EPSTI1 signal (Supplementary Fig. 7). Considering that resection samples were obtained 12–16 weeks after biopsies, these findings are consistent with therapy‑driven remodeling of the CRC TME and likely depletion of EPSTI1^+^ epithelial cell populations.

### JAK-STAT inhibition counteracts chemotherapy-induced ISG expression

To test whether pharmacologic JAK-STAT inhibition can suppress ISG expression, we treated HCT116 cells with ruxolitinib (0.5 µM) alone or in combination with fluorouracil (1 µM) or irinotecan (1 µM) and analyzed ISG expression up to 72 h. Ruxolitinib alone suppressed the expression of *EPSTI1*, *IFIT1*, *IRF9*, and *STAT1* over time, confirming pathway suppression at the transcriptional level. Consistent with stress-induced IFN signaling, fluorouracil and irinotecan modestly increased ISG expression. Notably, ruxolitinib counteracted this induction when combined with chemotherapy (Fig. 5I). Taken together, these findings support the scRNA-seq observations that chemotherapy augments ISG expression, including *EPSTI1*, and provide preliminary evidence that pharmacological inhibition of the JAK-STAT pathway counteracts therapy-induced gene expression and may be used to improve chemotherapy responses.

## Discussion

The molecular programs underlying the heterogeneity in epithelial cell-intrinsic responses to standard-of-care chemotherapy remain incompletely understood in CRC. Here, we combined drug response profiling of CRC PDTOs with bulk and single-cell transcriptomic analyses and functional assays to show that an epithelial ISG program, modulated by STAT1-regulated JAK-STAT signaling, is associated with reduced chemosensitivity. This ISG-high state is detectable in a subset of PDTOs at baseline and is further induced by fluorouracil and irinotecan, as evidenced by temporal STAT1 phosphorylation and chemotherapy-induced *EPSTI1* mRNA and protein expression. Together with our loss-of-function experiments showing that *EPSTI1* supports epithelial cell fitness and limits chemosensitivity, these results position epithelial ISG programs, and *EPSTI1* as an effector, as modulators of how CRC cells withstand genotoxic chemotherapy.

ISG expression in tumors is often attributed to IFN signaling driven by stromal or immune cells. Our observation that ISG expression persists in PDTOs devoid of immune components indicates that this transcriptional state can be maintained through an epithelial-intrinsic program. The ISG set enriched in low-chemosensitivity PDTOs shows substantial overlap with the IFN-related DNA damage resistance signature (IRDS), a program previously linked to resistance to radiotherapy and chemotherapy across multiple cancer types [41]. Although we did not directly measure IRDS activity or upstream regulators such as the U-ISGF3 complex, the sustained ISG expression without exogenous IFN, which is seen both in untreated PDTOs and in temporal profiling of CRC cell lines, points to a constitutive transcriptional state with potential stress-adaptive properties. In CRC, multiomic profiling has revealed a distinct inflammatory regenerative cancer cell (iREC) state characterized by ISG expression, which is thought to be induced by T cell-derived IFN signaling [42]. Our data suggest that similar ISG-associated cancer cell states can also arise and persist independently of immune cells, implying that immune-modulated and epithelial-intrinsic pathways may converge on a common, stress-adaptive program. A recent study utilizing lineage tracing and scRNA-seq defined a transcriptional state in breast cancer tumor-initiating cells characterized by IFN signaling and STAT1 activity, providing supporting evidence for our observations in CRC [43]. A comparable IFN-associated program has been also described in high-grade serous ovarian cancer, which is further enhanced by chemotherapy [44], similar to our observations from the scRNA-seq data of treatment-naïve and neoadjuvant therapy-exposed CRC tumors. Collectively, these findings support the hypothesis that intrinsic ISG maintenance may represent a cross-cancer adaptive program that enables cancer cells to tolerate chemotherapy, highlighting its potential as a therapeutic target.

While STAT1 signaling is generally considered to be a pro-apoptotic and tumor-suppressive, oncogenic role of *STAT1* has been described in *KRAS*-mutant CRC, linking *STAT1* to tumor growth [45] and chemotherapy resistance [46]. Our observations linking higher JAK-STAT pathway activity to reduced sensitivity to chemotherapy are consistent with the earlier observations suggestive of a tumor-promoting role.

Within this ISG-high program, *EPSTI1* emerged as a putative effector with a functional role in chemotherapy modulation. Earlier studies on *EPSTI1* have shown that *EPSTI1*, through ISG activation, may promote anti-tumor effects of multiple immune cell types and enhance responsiveness to immunotherapy. In addition to this potential immune biomarker role, *EPSTI1* expression in epithelial cancer cells is modulated by KSR1/Erk signaling axis, which is associated with the metastatic potential of cancer cells through the regulation of cell mobility and EMT-like phenotype [37]. Building on this foundation, our study expands the functional scope of *EPSTI1* by demonstrating its role in modulating drug response in CRC. Using complementary loss- and gain-of-function approaches, we show that *EPSTI1* depletion sensitizes CRC cells to standard-of-care chemotherapeutics, whereas transient overexpression confers partial drug tolerance. These findings position *EPSTI1* as a direct modulator of chemotherapy response, underscoring its potential as a therapeutic target within the broader ISG-related cancer cell state. We propose that *EPSTI1’s* apparently contrasting roles across different TME are not mutually exclusive but instead reflect its function under distinct biological conditions.

### Limitations of the study

The lack of a TME is a well-recognized limitation of PDTOs, and in vitro drug responses of epithelial tumor cells cannot fully capture the complexity of clinical responses in vivo. Recognizing that multiple cytokines that regulate STAT activity in the CRC TME are produced by immune and stromal cell types [47], the experimental models could not interrogate how such mediators contributed to drug responses in vitro. Large-scale tissue acquisition for characterizing primary tumors and establishing PDTOs mainly from surgical specimens may cause selection bias to focus only on tumors with incomplete pathological response, leading to underrepresentation of tumor subtypes with high chemosensitivity. Another caveat is culture-associated transcriptional remodeling, a phenomenon that occurs over long-term culture [48], which may confound DEA and GSEA results. Finally, mechanistic insights are derived from functional validation of *EPSTI1* but not the broader ISG program. Future work integrating temporal gene expression profiling in larger PDTO cohorts and systematic perturbations of ISG pathway activity will be essential to fully describe the dynamic transcriptional programs underlying chemotherapy response.

## Supplementary information

Supplementary Material

Supplementary Table 1

Supplementary Table 2

Supplementary Table 3

Supplementary Table 4

## Data Availability

Processed bulk RNA-seq was deposited in the Gene Expression Omnibus (GEO, accession number: GSE292601) and made publicly available at the time of publication. All computational codes were deposited in Zenodo (https://doi.org/10.5281/zenodo.15053583) and made publicly available at the time of publication. Additional information required to reanalyze the data will be provided by Erdogan Pekcan Erkan (erdogan.erkan@tuni.fi). Requests for materials or reagents should be directed to the lead contact Toni Seppälä (toni.seppala@tuni.fi).
