# Supplementary material for: Epithelial interferon-stimulated gene programs modulate chemotherapy sensitivity in colorectal cancer via EPSTI1
Source: Oncogene. 2026 Aug 21;45(41):4377–92. doi: 10.1038/s41388-026-03957-9 (PMC13645637; doi:10.1038/s41388-026-03957-9)
Supplement: Supplementary file 1 [file 41388_2026_3957_MOESM1_ESM.pdf]

SUPPLEMENTARY MATERIAL

List of Supplementary Tables

Supplementary tables are given as separate files.

Supplementary Table 1. Molecular classification of PDTOs.

Supplementary Table 2. Aggregated GSEA results.

Supplementary Table 3. Sequence of primers used in RT-qPCR.

Supplementary Table 4. Full list of authors in the iCAN consortium.

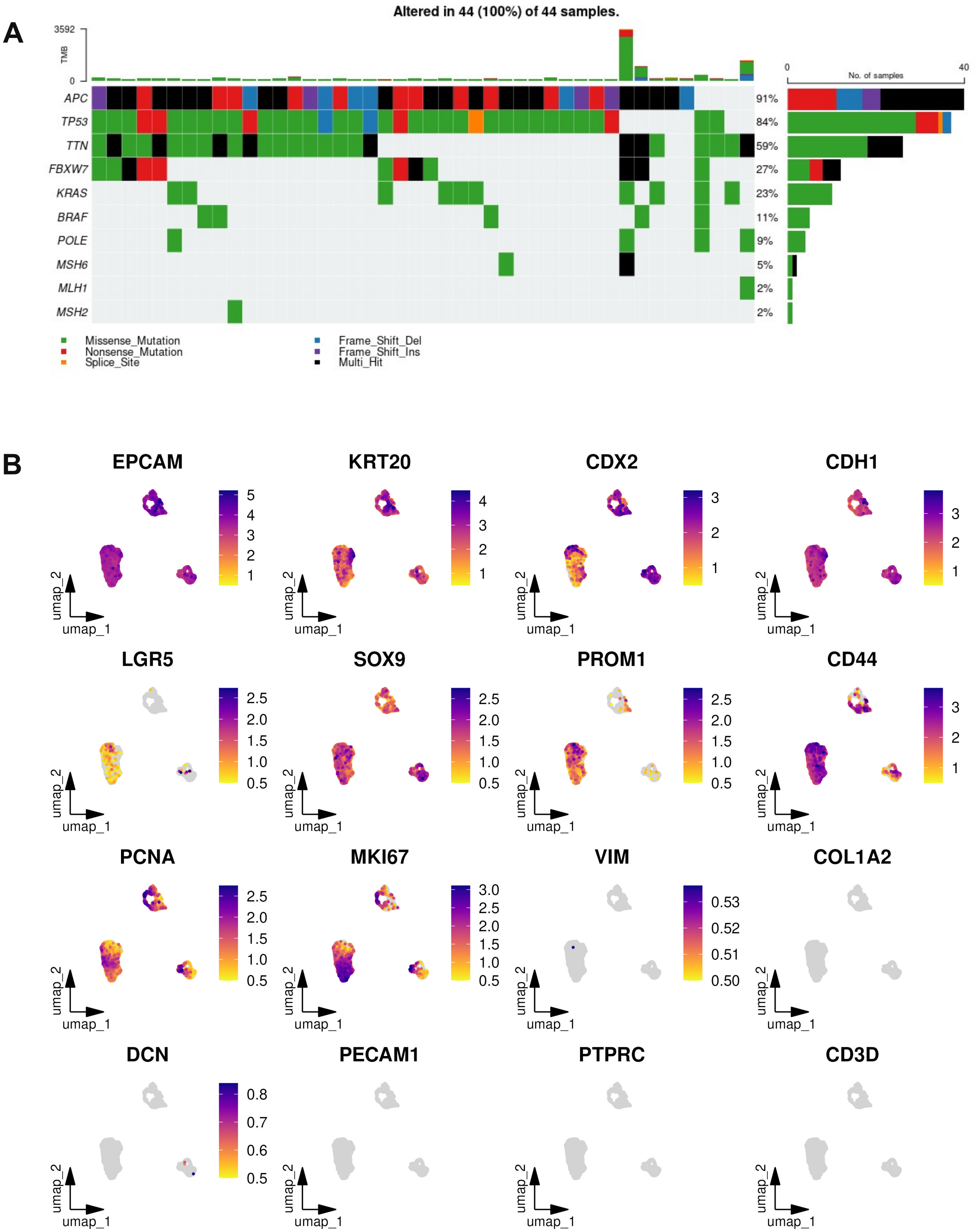

Supplementary Figure 1. Oncoplot showing CRC-related driver mutations across 44 PDTOs (A). Feature plots showing epithelial-, stem-like, stromal-, and immune-related gene expression in PDTOs (B).

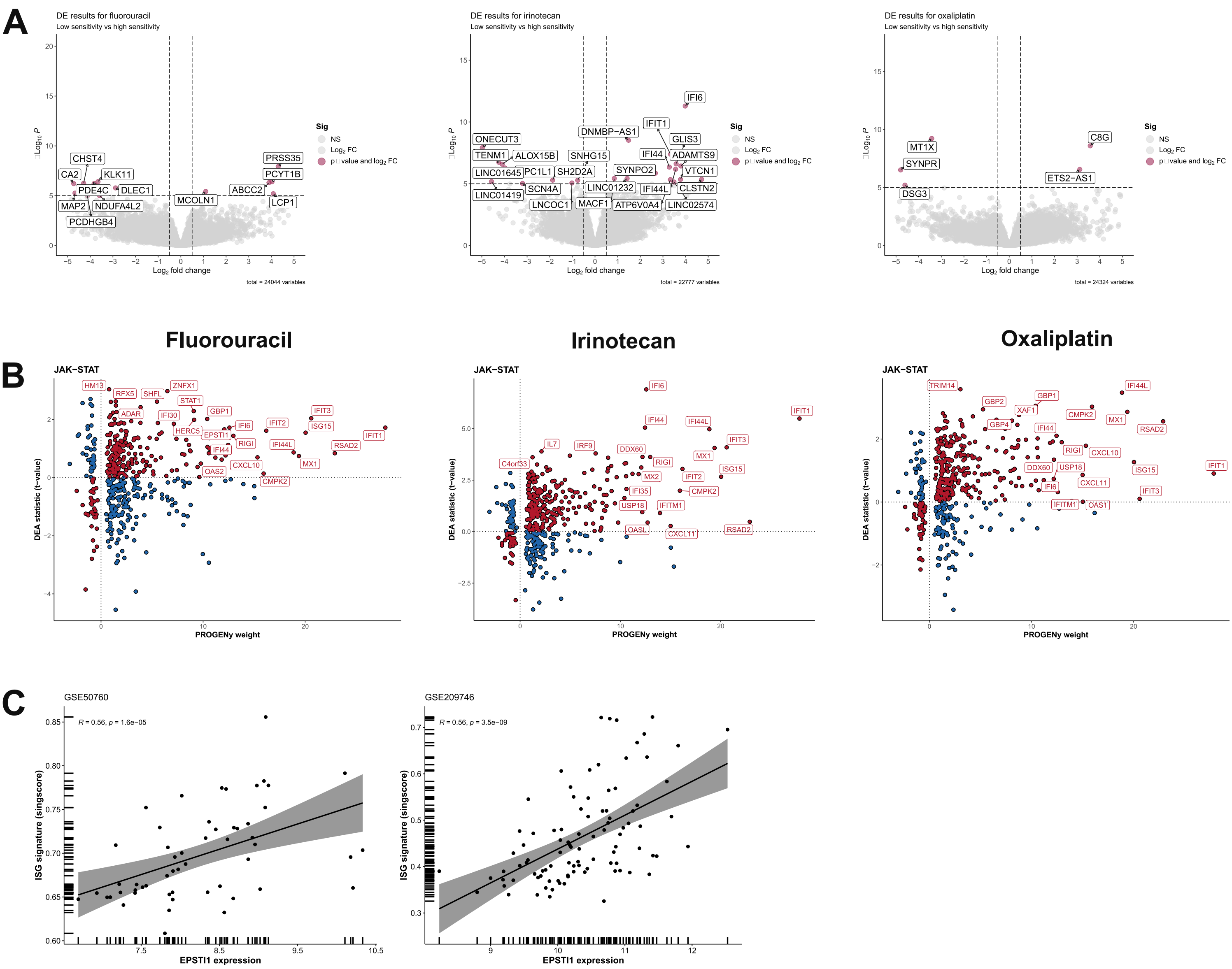

Supplementary Figure 2. Volcano plots showing the DEA results for each drug (A). Scatter plots showing the relationship between JAK-STAT target gene weights and corresponding t-values calculated in DEA for each comparison (B). Scatter plots showing the correlation between EPSTI1 expression and ISG module score in GSE50760 and GSE209746 datasets (C).

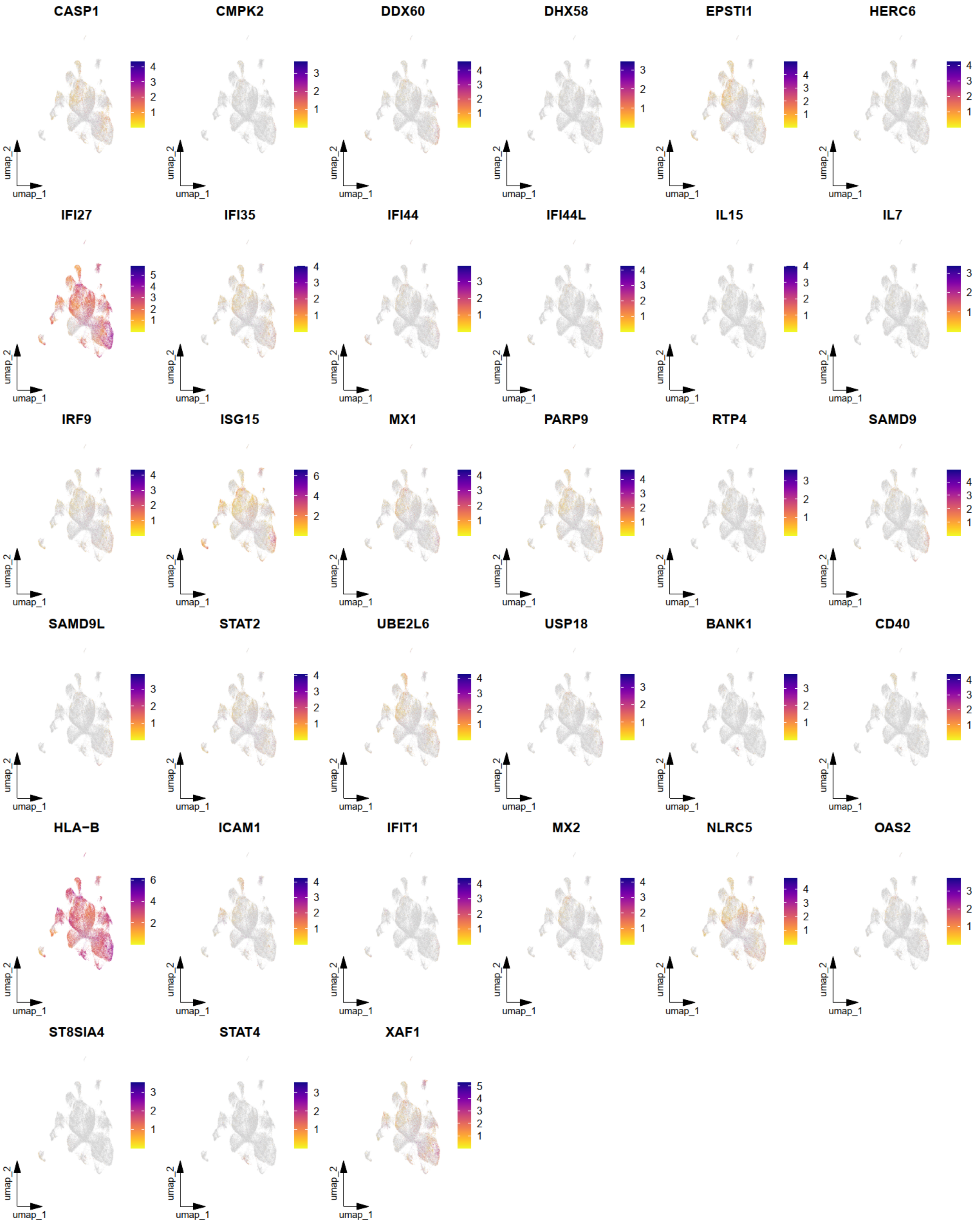

Supplementary Figure 3. Feature plots showing the expression of ISGs in the CNP0004138 scRNA-seq dataset.

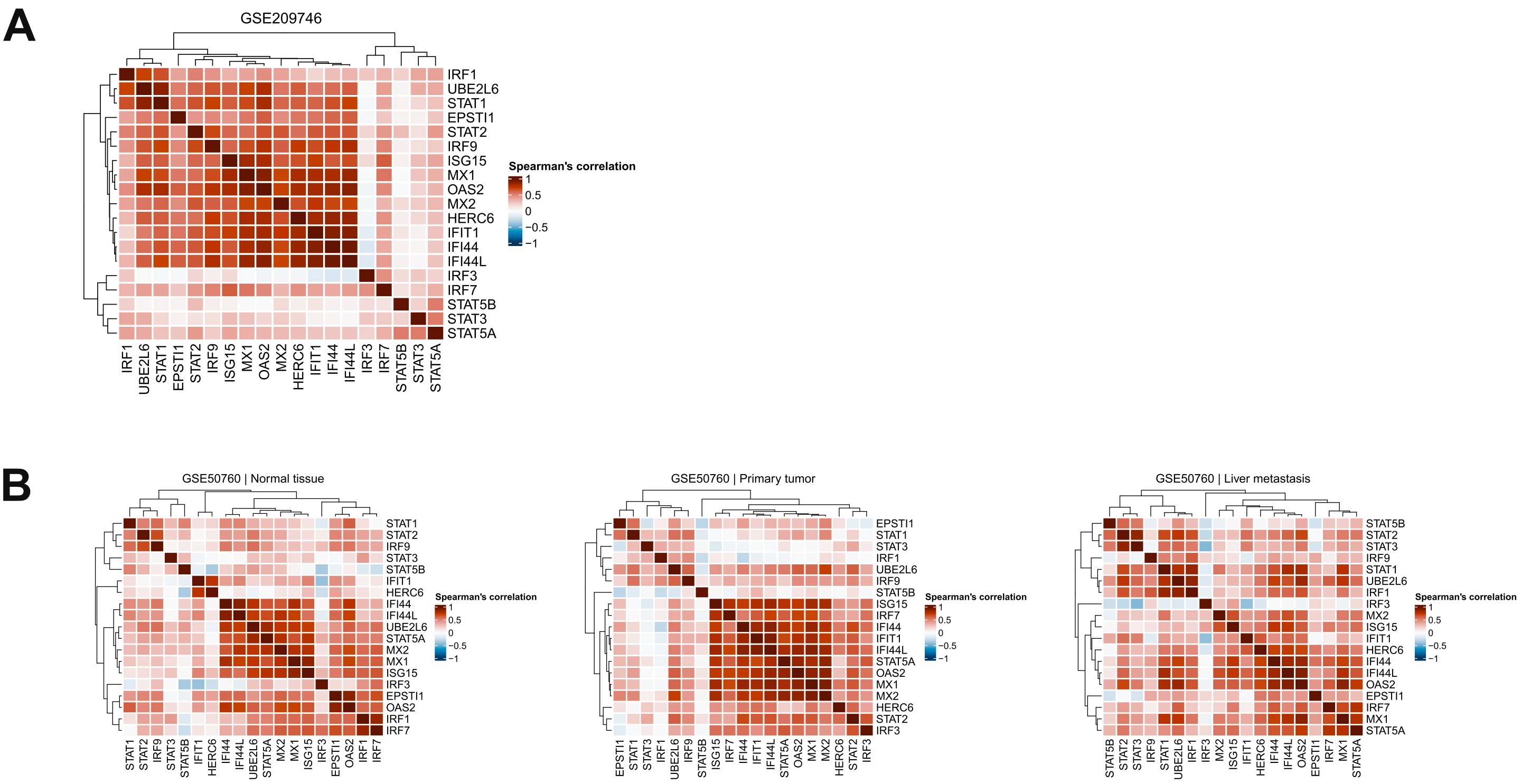

Supplementary Figure 4. STAT family involvement in ISG programs. Heatmap of Spearman gene-gene correlations between STAT family members and representative ISGs in the bulk RNA seq dataset GSE209746 (A). Heatmaps of the same STAT-ISG gene set in the bulk RNA seq dataset GSE50760, shown separately for normal colon, primary tumor, and liver metastasis samples (B).

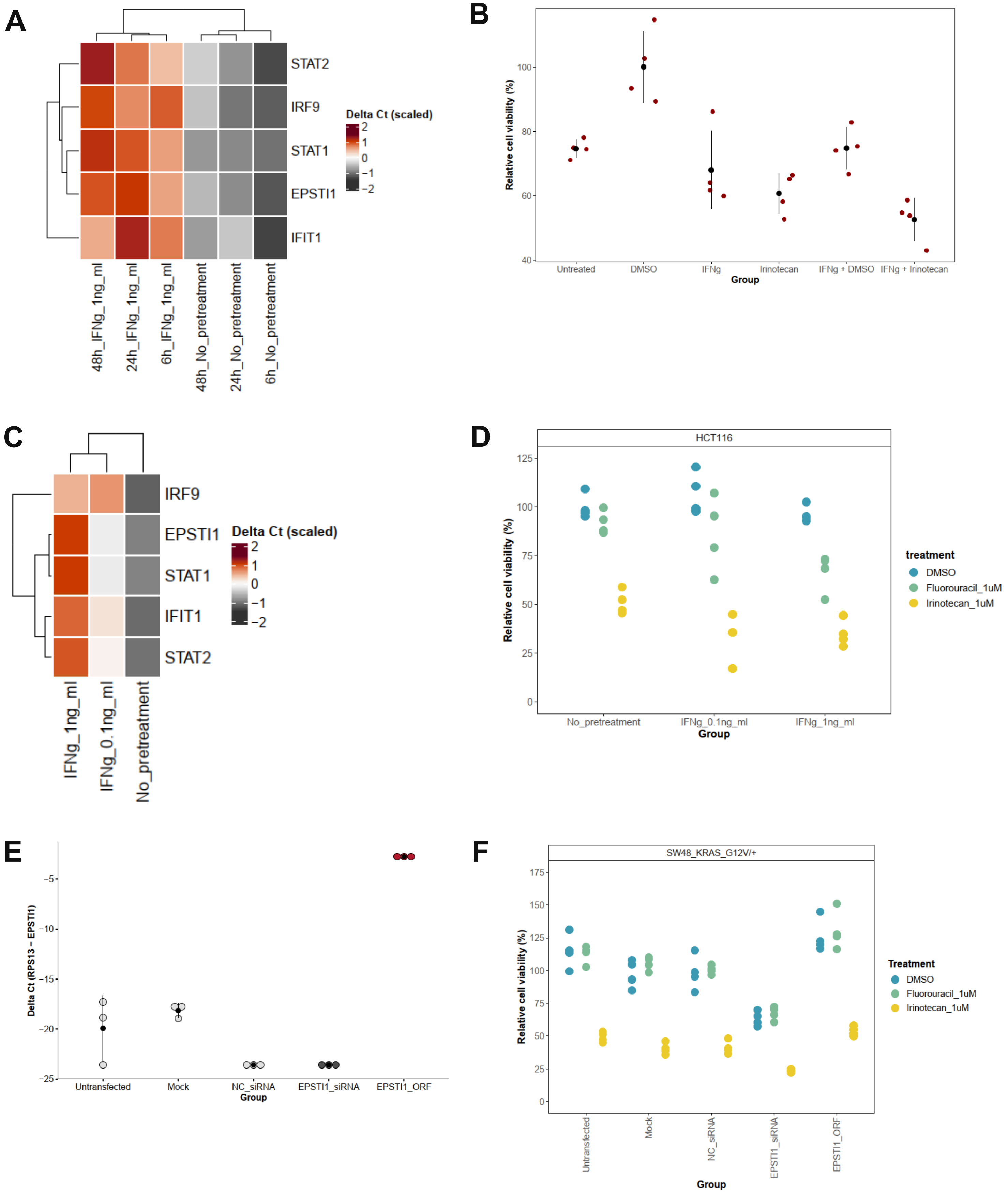

Supplementary Figure 5. Short-term (24 h) pretreatment with 1 ng/mL IFN gamma-induced ISG expression in HCT116 cells in a time-dependent manner (A). Despite IFN gamma-induced ISG expression, IFN gamma-irinotecan combination was marginally more cytotoxic than irinotecan alone (B). Prolonged pretreatment (7 d) with a lower dose of IFN gamma (0.1 ng/mL) was able to induce ISG expression (C). Prolonged pretreatment also failed to reduce chemosensitivity and remained marginally cytotoxic (D). Validation of EPSTI1 overexpression in SW48 KRAS G12V/+ cells. EPSTI1 knockdown could not be reliably confirmed due to low baseline EPSTI1 expression (E). EPSTI1 knockdown and overexpression in SW48 KRAS G12V/+ cells resulted in chemosensitivity phenotypes similar to those observed in Caco-2 cells (F).

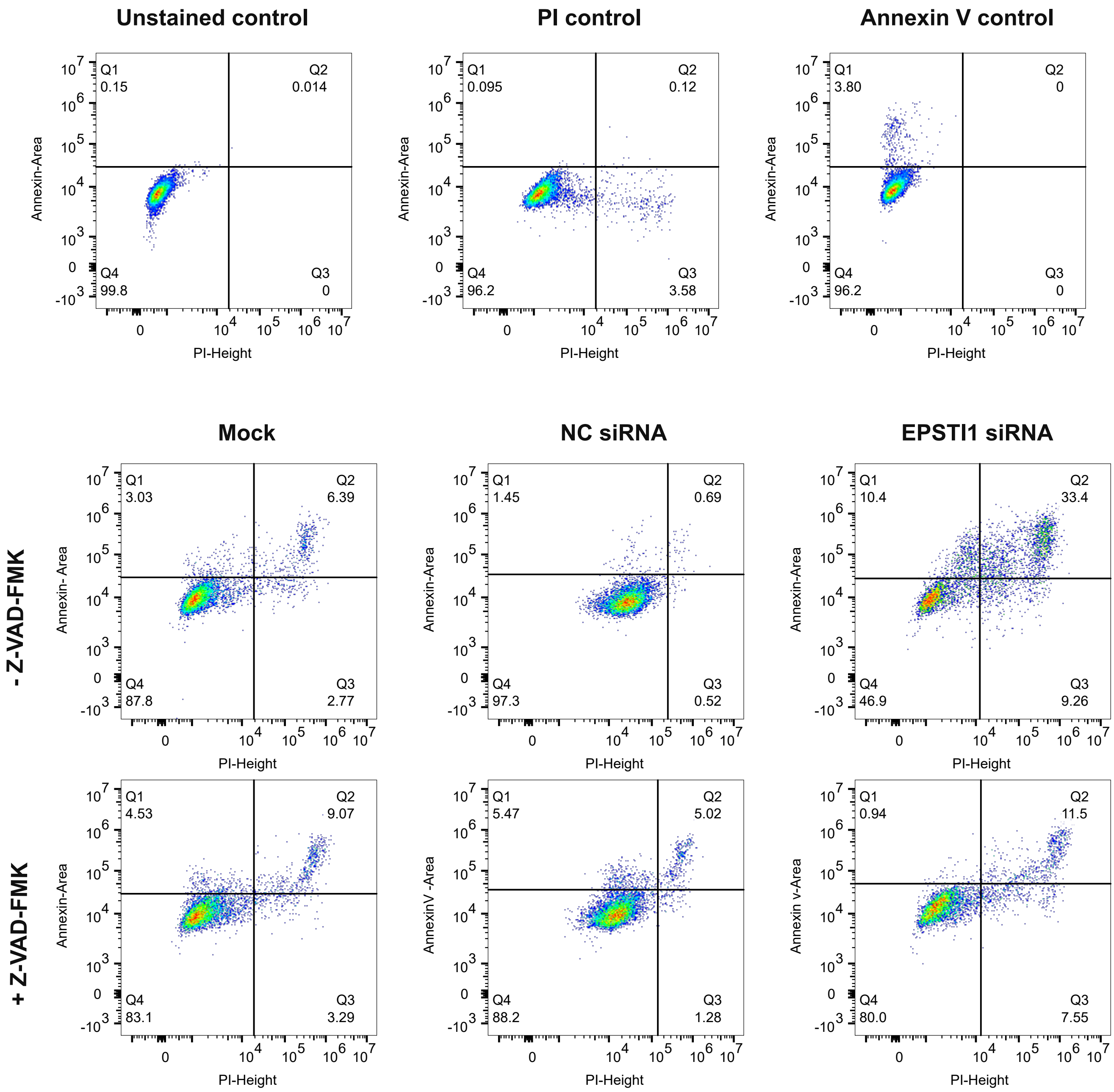

Supplementary Figure 6. Representative flow cytometry plots showing Annexin V/propidium iodide (PI) staining. In each experimental group, the percentage of single cells in each quadrant are shown. Q1: Annexin V$^+$/PI$^-$ (early apoptotic); Q2: Annexin V$^+$/PI$^+$ (late apoptotic); Q3: Annexin V$^-$/PI$^+$(necrotic); Q4: Annexin V$^-$/PI$^-$ (viable).

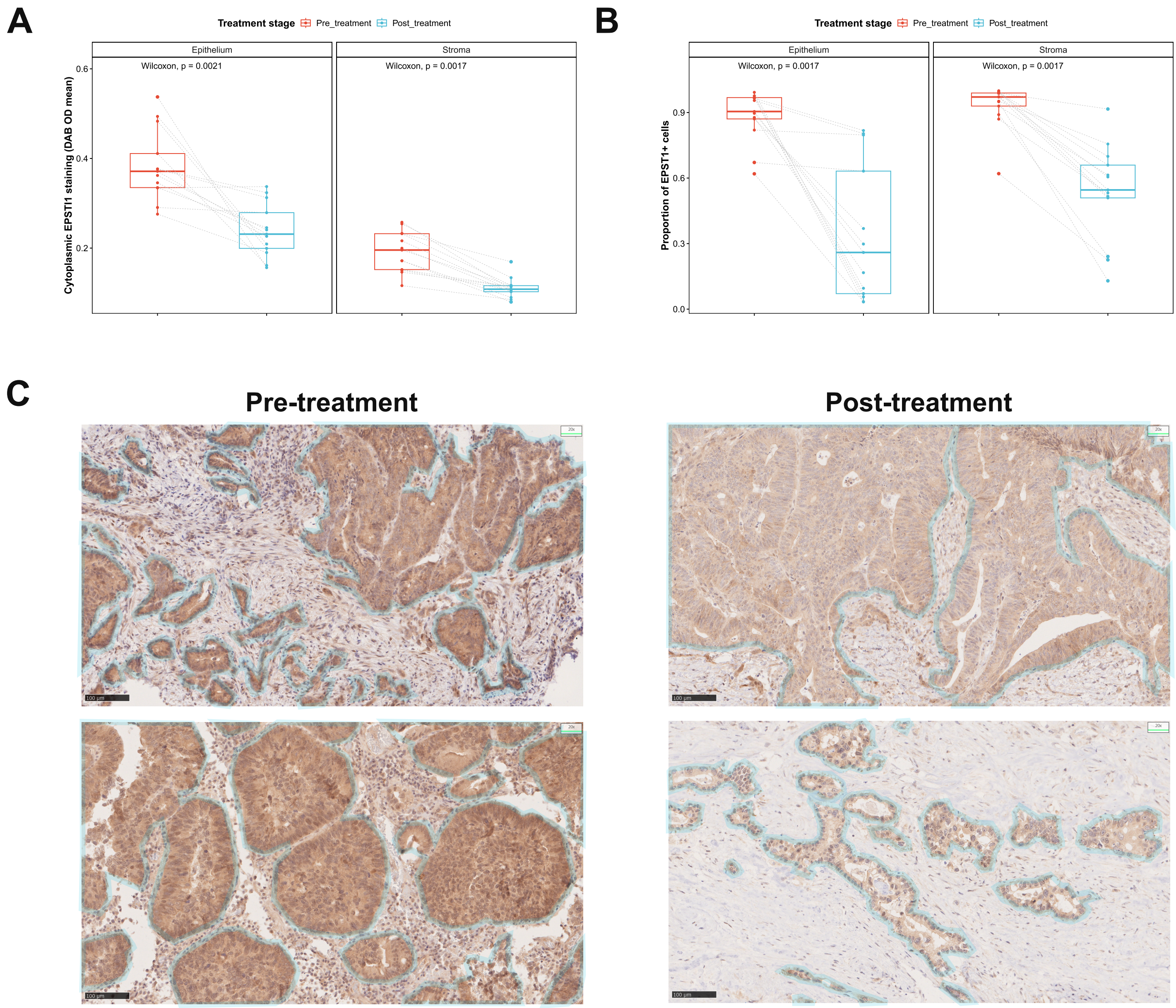

Supplementary Figure 7. Chemotherapy-induced changes in EPSTI1 protein expression in the CRC TME. Immunohistochemistry was performed for EPSTI1 in patient-matched pre-treatment biopsies and post-treatment resections.Box plots show reduction of cytoplasmic EPSTI1 staining (A) and the fraction of EPSTI1^{+} epithelial cells in post-treatment samples (B). Representative images taken from pre- and post-treatment samples show EPSTI1 expression in tumor (teal outline) and surrounding stroma (C).
